# Extracellular vesicles at the immune–metabolic crossroads of Hashimoto’s thyroiditis and diabetes mellitus

**DOI:** 10.3389/fimmu.2026.1914528

**Published:** 2026-08-19

**Authors:** Yao Ma, Wei-na Jiang, Tong Zhou, Bo Sun, Cheng-jun Gong, Le Guan, Qing-feng Wang

**Affiliations:** 1 Liaoning University of Traditional Chinese Medicine, Shenyang, China; 2 Liaoning Provincial Academy of Traditional Chinese Medicine, Shenyang, China; 3 The Second Hospital Affiliated to Liaoning University of Traditional Chinese Medicine, Shenyang, China; 4 The Affiliated Hospital of Liaoning University of Traditional Chinese Medicine, Shenyang, China

**Keywords:** diabetes mellitus, extracellular vesicles, Hashimoto’s thyroiditis, immune microenvironment, microRNA, thyro-metaflammation

## Abstract

Hashimoto’s thyroiditis (HT) and diabetes mellitus are highly prevalent chronic immune-mediated disorders that frequently co-occur and share genetic susceptibility, T-helper (Th) 1/Th17 skewing, and regulatory T-cell (Treg) dysfunction. Among individuals with type 1 diabetes mellitus (T1DM), autoimmune thyroiditis is the most common comorbid autoimmune disease. Extracellular vesicles (EVs) have emerged as important mediators linking autoimmune and metabolic inflammation. This review compares how EVs remodel the immune microenvironment in HT, type 2 diabetes mellitus (T2DM), and related disease contexts, with attention to donor cells, cargo, recipient pathways, biomarkers, and therapeutic implications. In HT and T1DM, EVs can deliver organ-specific autoantigens, whereas in classical T2DM current evidence more strongly supports EVs as carriers of stress signals, chemokines, and immunoregulatory miRNAs that shape islet inflammation and insulin resistance rather than autoantigen presentation; latent autoimmune diabetes in adults is considered separately. Across these diseases, recurrent EV-miRNA programs and DAMP/NLRP3 signaling converge on Treg/Th17 imbalance and M1/M2 macrophage polarization. We also emphasize the marked asymmetry of evidence maturity, with substantially stronger *in-vivo* and clinical support on the T2DM side than on the HT side. This asymmetry is treated as an explicit interpretive boundary throughout the review. We assess circulating and urinary EV cargoes as liquid-biopsy candidates and discuss EV-based drug delivery, engineered immunomodulatory EVs, and modulation of EV biogenesis. Translational claims remain limited by heterogeneity, manufacturing, and safety challenges, particularly in organ-specific autoimmune disease. In this review, the term “immune–metabolic crossroads” refers to shared mechanisms, shared biomarker opportunities, and partially overlapping therapeutic entry points.

## 1 Introduction

Thyroiditis and diabetes mellitus are two common chronic immune-mediated diseases with a rising global burden. Hashimoto’s thyroiditis (HT) is the most prevalent form of autoimmune thyroid disease (AITD). A systematic review and meta-analysis of 48 studies estimates the global adult HT prevalence at 7.5% (95% CI, 5.7%–9.6%), with marked geographic and sex-related differences (1). The burden of diabetes is comparable. According to the 11th edition of the International Diabetes Federation (IDF) Diabetes Atlas, approximately 589 million adults aged 20–79 years live with diabetes in 2024, a figure projected to reach about 852 million by 2050 (2).

The two conditions frequently co-occur. Among patients with T1DM, the prevalence of concurrent autoimmune thyroiditis ranges from 5.5% to 41.2%, making it the most common comorbid autoimmune disease in this population (3). HT and T1DM share genetic susceptibility loci such as HLA-DR3, PTPN22 and CTLA-4, and both involve Th1/Th17 imbalance together with regulatory T-cell (Treg) dysfunction (4). This tight immunological overlap suggests a shared immunoregulatory network and warrants systematic mechanistic analysis from new perspectives.

**Figure 1:**
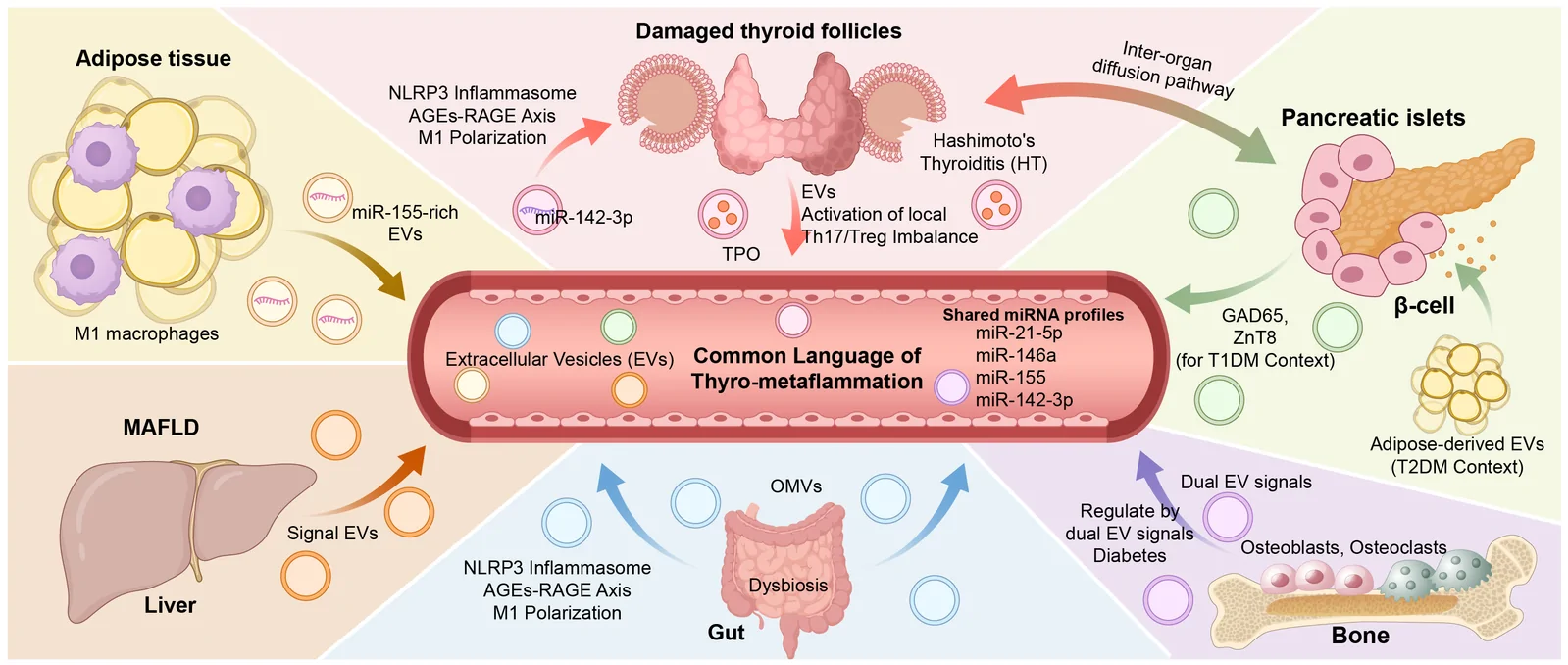
Extracellular vesicles constitute the common molecular language of thyro-metaflammation bridging Hashimoto's thyroiditis and type 2 diabetes mellitus. EV biogenesis inset. The inset schematizes the three principal EV subclasses that populate the systemic circulation depicted in the main image. Exosomes (30-150 nm; markers CD9, CD63, CD81, TSG101, Alix) originate from inward budding of the endosomal membrane to form multivesicular bodies (MVBs), which subsequently fuse with the plasma membrane to release intraluminal vesicles into the extracellular space; cargo sorting proceeds through both ESCRT-dependent and ESCRT-independent (ceramide/nSMase2, tetraspanin-mediated) pathways. Microvesicles (100-1000 nm) bud directly outward from the plasma membrane. Apoptotic bodies (>1000 nm) are generated by membrane blebbing of cells undergoing programmed cell death. Arrows in the inset distinguish the cargo-loading routes and the release step of each subclass. This inset replaces the discursive biogenesis description previously presented in Section 4.1. Systemic circuit (main image). Multiple endocrine and immune-metabolic compartments, including M1-polarized adipose tissue macrophages, MAFLD-affected liver, dysbiotic gut, bone-derived osteoblast/osteoclast units, and pancreatic beta-cells, release EVs into the systemic circulation. These vesicles converge on a shared cargo signature dominated by miR-21-5p, miR-146a, miR-155, and miR-142-3p, together with tissue-specific autoantigens (TPO for HT; GAD65 and ZnT8 for T1DM). Through the inter-organ diffusion pathway, EVs deliver NLRP3 inflammasome activators, AGEs-RAGE-axis signals, and M1-polarizing cues to damaged thyroid follicles, thereby activating local Th17/Treg imbalance and HT-type follicular destruction. The same EV pool simultaneously perturbs beta-cell function and shapes islet and peripheral tissue inflammation in T2DM through delivery of stress cargoes, chemokines, and immunoregulatory miRNAs rather than through autoantigen presentation, establishing a bidirectional immune-metabolic communication loop consistent with the evidence boundaries stated in Sections 2.3.1 and 9. Pathways depicted in this image are constructed from the following primary evidence anchors: EV biogenesis, cargo sorting, and MISEV2023-compliant subclass definitions (6, 7); thyro-metaflammation and shared immune microenvironment axes in HT and T2DM (8-12); inter-organ EV signaling between adipose tissue, liver, gut, bone, thyroid, and pancreatic islets (8, 13); and NLRP3 and AGEs-RAGE amplification in the shared inflammatory node (references retained as in the corresponding Sections 3 and 6 citations). This image remains a fully original schematic and does not reproduce any published pathway diagram; the citations above anchor the mechanistic content of the image to the source literature. AGEs, advanced glycation end-products; Alix, ALG-2-interacting protein X; CD9/CD63/CD81, tetraspanin exosome markers; ESCRT, endosomal sorting complex required for transport; EVs, extracellular vesicles; GAD65, glutamic acid decarboxylase 65; HT, Hashimoto's thyroiditis; MAFLD, metabolic dysfunction-associated fatty liver disease; M1, classically activated (pro-inflammatory) macrophage phenotype; miRNA, microRNA; MVB, multivesicular body; nSMase2, neutral sphingomyelinase 2; NLRP3, NLR family pyrin domain containing 3; RAGE, receptor for advanced glycation end-products; T1DM, type 1 diabetes mellitus; T2DM, type 2 diabetes mellitus; Th17, T-helper 17 cell; TPO, thyroid peroxidase; Treg, regulatory T cell; TSG101, tumor susceptibility gene 101; *ZnT8, zinc transporter 8*.

Extracellular vesicles (EVs) are non-replicating lipid-bilayer particles released by virtually all cell types under physiological and pathological conditions. Major subclasses include exosomes (30–150 nm), which arise from multivesicular bodies; microvesicles (100–1000 nm), which bud directly from the plasma membrane; and apoptotic bodies (>1000 nm), which are generated during programmed cell death (5). Cargo loading can proceed through ESCRT-dependent or ESCRT-independent routes, and the MISEV2023 framework recommends operational nomenclature based on physical characteristics such as size rather than presumed biogenesis alone (6). By transferring miRNAs, proteins, and lipids to recipient cells, EVs participate in intercellular communication and immune regulation; EV biogenesis and cargo-loading are summarized schematically in **Figure 1** (5, 14).

In autoimmune disease, EVs present autoantigens and modulate immune-cell function, disrupting immune tolerance (15). In HT thyroid tissue, T-lymphocyte–derived tissue small EVs enriched in miR-142-3p impair Treg function and accelerate destruction of thyroid follicular cells (8). In T1DM, β-cells under endoplasmic reticulum stress release EVs carrying islet autoantigens such as GAD65 and IA-2, and their uptake by professional antigen-presenting cells helps to prime islet-reactive T cells—a mechanism directly documented in T1DM (13). In classical T2DM, by contrast, EVs from β-cells and other cellular sources are best supported as carriers of stress signals, chemokines (e.g., CXCL10) and immunoregulatory miRNAs that shape islet inflammation, macrophage polarization and systemic insulin resistance, rather than as autoantigen-presenting platforms (9). This mechanistic boundary between T1DM and T2DM is maintained throughout the review. EVs are thus emerging as a bridge between autoimmune and metabolic-disease immunology.

Current EV research in HT and diabetes remains largely disease-specific, and direct comparison of immune-microenvironment remodeling across the two conditions is still limited. Yet their frequent comorbidity and shared immune features make such a comparison clinically and biologically relevant. EVs are also attractive as liquid-biopsy substrates for disease detection and monitoring (16). Preclinical studies further suggest therapeutic potential, including mesenchymal stem cell (MSC)–derived EVs in T1DM and surface-engineered EVs displaying immune-checkpoint molecules such as PD-L1 (17, 18); however, these translational prospects must be interpreted cautiously because engineered EVs still face substantial challenges in batch consistency, *in-vivo* clearance, and off-target immune activation, particularly in organ-specific autoimmune disease such as HT (5).

This review examines how EVs reshape the immune microenvironment in HT and diabetes, compares shared and disease-specific mechanisms, and evaluates their relevance as biomarkers and therapeutic targets. Throughout the manuscript, “immune–metabolic crossroads” is used in a defined three-layer sense: shared EV-mediated mechanisms, shared biomarker opportunities, and shared therapeutic entry points. The strength of evidence is not uniform across these layers or across diseases (5). In particular, EV research in T2DM includes substantially more *in-vivo* functional work, transgenic models, and early clinical data than EV research in HT, which remains dominated by cellular models and small-sample clinical correlation studies. That asymmetry is stated up front and used as an interpretive boundary throughout the review; mechanisms supported on the HT side only by correlative observation and lacking functional cargo-transfer evidence are identified as extrapolated from T2DM-side evidence.

## 2 Extracellular vesicles: molecular carriers bridging the immune–metabolic crossroads of Hashimoto’s thyroiditis and type 2 diabetes

Hashimoto’s thyroiditis (HT) is classically defined as an organ-specific autoimmune disease, whereas T2DM is regarded as a chronic metabolic disorder. These two conditions are traditionally placed in distinct nosological categories. Yet epidemiological data consistently show that their co-occurrence is not coincidental. The prevalence of HT is significantly higher in T2DM populations than in the general population (1). A meta-analysis of prospective observational studies has confirmed a significant association between thyroid dysfunction and the risk of T2DM (19). This systemic comorbidity cannot be explained by a single endocrine axis and points to a deeper shared immune–metabolic communication mechanism between HT and T2DM.

This Section establishes a transverse comparative framework for HT and T2DM based on the comparability of EVs across the “biogenesis–loading–delivery–response” continuum. The central thesis is twofold: EVs are capable of carrying autoantigens and immunoregulatory signals in autoimmune contexts (20), and they simultaneously transport β-cell stress signals, chemokines and immunoregulatory miRNAs that shape metabolic inflammation and insulin resistance in T2DM (9). This dual capacity—autoimmune signal transport on the one hand and metabolic-inflammatory signal transport on the other—positions EVs as the key molecular carriers linking the two diseases, while preserving the mechanistic boundary between antigen-driven and metabolism-driven pathology.

### 2.1 A note on asymmetric evidence maturity (moved forward)

The two disease fields show marked asymmetry in EV-related evidence maturity, and we state this caveat at the outset of Section 2 so that every subsequent cross-disease inference is read against it. T2DM-related EV research has accumulated substantial *in-vivo* functional validation, transgenic models and early clinical evidence (9). By contrast, EV research in HT remains dominated by cellular models and small-sample clinical correlation studies, with limited *in-vivo* functional evidence (20). This asymmetry requires a cautious analytical strategy: wherever a mechanism is supported only by correlative observation on the HT side and lacks functional cargo-transfer evidence, the review explicitly labels the inference as “extrapolated from T2DM-side evidence.” Existing evidence gaps and feasible verification pathways are discussed systematically in Section 8. When evaluating individual studies, this review adheres to the minimal reporting standards established by MISEV2023 (6); studies that explicitly identify the EV donor cell source and provide functional cargo-transfer evidence are given priority.

### 2.2 Rationale: the shared immune–metabolic interface of HT and T2DM

Three layers of evidence support the use of EVs as a unified entry point for comparing HT and T2DM.

First, the two diseases share a comparable pattern of immune microenvironment imbalance. In immunological terms, this pattern refers to a coordinated shift away from FOXP3^+^ regulatory T-cell (Treg)–mediated tolerance toward Th1- and Th17-driven inflammatory effector responses, together with M1-skewed macrophage polarization. In HT, the thyroid infiltrate is dominated by expansion of CD4^+^ Th1/Th17 cells and progressive Treg attrition (10, 12). In T2DM, the pancreatic islet microenvironment is dominated by M1-like macrophages, while systemic adipose tissue shows the same M1 skewing that drives low-grade chronic inflammation (11). Across both conditions, the shared failure node is Treg differentiation and FOXP3 stability (12) — a convergence that provides the immunological rationale for treating the two diseases within one EV-centered framework.

Second, the two diseases share a common core panel of pro-inflammatory cytokines, including TNF-α, IL-6, IL-1β and IFN-γ. These mediators disrupt immune homeostasis within thyroid tissue (21) while simultaneously impairing insulin signaling in peripheral tissues (22).

Third, EVs have been recognized as “pathogenic carriers” in multiple systemic autoimmune diseases (15), and a parallel role for EVs has been systematically summarized in T2DM and its complications (9). These two independent evidence streams together support the unified treatment of EVs as the molecular carrier of this Section.

### 2.3 Shared donor cells: a bidirectional loop between endocrine target cells and infiltrating immune cells

#### 2.3.1 EVs from endocrine target cells: parallel features of thyroid follicular cells and pancreatic β-cells

Thyroid follicular cells (TFCs) and pancreatic β-cells are both highly secretory endocrine cells with well-developed rough endoplasmic reticulum and an active secretory vesicle pathway. Under pathological conditions, both cell types upregulate EV release and remodel their cargo profile (14).

In HT patients, serum exosomes are enriched in TPO, Tg, HMGB1, HSP60 and MHC-II. These exosomes are taken up by DCs and activate DCs through TLR2/3–NF-κB signaling, inducing imbalanced CD4^+^ T-cell differentiation (23). *In vitro* evidence shows that thyrocyte-derived exosomes directly target DCs and elicit a stronger CD4^+^ T-lymphocyte response (24).

On the β-cell side, inflammatory and endoplasmic-reticulum stress markedly reshapes the EV composition of insulin-secreting cells. In T1DM, the resulting EVs carry islet-specific autoantigens (GAD65, IA-2, ZnT8, proinsulin) and function as antigen-bearing signals, whereas in T2DM the resulting EVs are best supported as carriers of stress-response molecules, chemokines and immunoregulatory miRNAs that amplify low-grade islet inflammation without engaging a classical autoantigen-presentation route (9, 13). This section deliberately keeps the T1DM antigen-presentation mechanism separate from the T2DM metabolic-inflammatory mechanism.

The common outcome of EV release from these two cell types is the conversion of local “cellular stress events” into molecular signals readable by distant immune cells.

#### 2.3.2 EVs from infiltrating immune cells: a cross-disease mechanism of immune signal homologation

Infiltrating immune cells also act as active EV donors that reshape the local microenvironment.

Within HT thyroid infiltrates, T-lymphocyte–derived small EVs are enriched in miR-142-3p. Internalization of this miRNA by Treg cells induces Treg dysfunction and downregulates the RAC1/ERK1/2 pathway, thereby aggravating thyrocyte destruction. This represents one of the few functional EV cargo-transfer evidences on the HT side (8).

In T2DM-related obesity models, small EVs secreted by adipose tissue macrophages modulate systemic insulin sensitivity through miRNA cargo and can directly mediate insulin sensitization (25).

Cross-disease comparison reveals concordant directional shifts: protective Treg-associated miRNAs such as miR-146a are downregulated in both conditions (26), while destructive cargo in effector immune-cell–derived EVs is concurrently upregulated (27). This directional convergence indicates that the two diseases share the same imbalance pattern at the immune–EV level.

### 2.4 Shared immunoactive cargo: autoantigens, miRNAs and DAMPs

#### 2.4.1 Autoantigen cargo: a mechanism distinctly documented in HT and T1DM, and a bystander signal in T2DM

EVs can carry membrane-bound MHC–peptide complexes or intracellular autoantigenic proteins. This property allows EVs to function as “mobile antigen-presenting units” that bypass the classical contact-dependent antigen presentation route (7).

In HT, circulating exosomes carry TPO, Tg and HLA-II molecules. After uptake by DCs, these exosomes activate imbalanced CD4^+^ T-lymphocyte responses, constituting a critical initiating event of HT (23).

In T1DM, β-cell–derived EVs carry the islet-specific autoantigens GAD65, IA-2, ZnT8 and proinsulin. Under endoplasmic reticulum stress and pro-inflammatory cytokine exposure, the release of these autoantigen-bearing EVs is markedly increased, and their uptake by professional antigen-presenting cells in the pancreatic lymph nodes primes islet-reactive T cells—a mechanism directly documented in T1DM models and in the reference we now cite (13, 28).

In contrast, T2DM is not classically an autoantigen-driven disease, and we do not extend the T1DM antigen-presentation mechanism to T2DM. What is recognized in T2DM is the existence of an autoimmune subgroup—latent autoimmune diabetes in adults (LADA)—in whom islet autoantibodies (GADA, IA-2A, ZnT8A) are detectable and disease progression shows features intermediate between T1DM and classical T2DM; whether β-cell EVs contribute to autoantibody generation in LADA remains hypothesis-generating and requires dedicated evidence. In classical T2DM, β-cell EV cargo is best supported as a carrier of stress signals, chemokines (e.g., CXCL10) and immunoregulatory miRNAs that shape islet inflammation and insulin resistance, rather than as an autoantigen-presenting platform (9).

When HT co-occurs with T1DM, the simultaneous release of autoantigen-bearing EVs from two endocrine organs may plausibly reinforce a shared tolerance-breakdown loop; extension of this synergy hypothesis to HT–T2DM comorbidity is not supported by current evidence and is not claimed here.

#### 2.4.2 EV-miRNA: a common mechanism regulating Treg/Th17 and M1/M2 polarization

miRNAs are the most functionally well-characterized transferrable cargo within EVs. They silence target genes at the post-transcriptional level within recipient cells and thereby reprogram recipient cell phenotype (29). Cross-comparison of HT and T2DM literature reveals a recurring set of key EV-miRNAs.

miR-142-3p. This miRNA is enriched in T-cell–derived tissue small EVs in HT thyroid and induces Treg dysfunction together with thyrocyte destruction (8).
miR-155 and miR-146a. Both are listed as key dysregulated miRNAs participating in immune imbalance in AITD (30) and are also abnormally expressed in T2DM-related metabolic inflammation (26).
Immunoregulatory molecules carried by β-cell EVs. Beyond miRNAs, β-cell EVs also carry immune checkpoint molecules such as PD-L1, which regulate CD8^+^ T-cell activity and serve as potential biomarkers during T1DM progression (31).

The central insight from these observations is that, despite differing target organs and modes of onset, the downstream pathways mediated by EV-miRNAs in HT and T2DM ultimately converge on Treg/Th17 imbalance and M1/M2 polarization imbalance.

#### 2.4.3 DAMPs and the amplification loop of “sterile inflammation”

EVs released under stress can be enriched in DAMPs and mitochondria-derived components. In an acute pancreatitis model, M1 macrophage–derived EVs deliver inflammatory mitochondria into pancreatic β-cells and induce ferroptosis. This evidence demonstrates a non-classical mechanism by which EVs mediate cross-organ inflammatory–metabolic injury (32).

This mechanism provides a molecular-level explanation for the “persistent low-grade inflammation” phenotype shared by HT and T2DM. Both diseases lack a clear exogenous infectious trigger yet maintain chronic elevation of systemic inflammatory markers. To visualize the donor–cargo–recipient–outcome relationships across HT, T1DM and T2DM that have been described in this section, the principal experimental evidence is summarized in **Table 1**.

**Table 1:** Extracellular vesicle cargo, donor–recipient cells, and immunological outcomes in Hashimoto’s thyroiditis and diabetes mellitus.

Disease	Donor cell	Major EV cargo	Recipient cell	Signaling pathway	Immunological/pathological outcome	Ref.
HT	Thyroid follicular cells	TPO, Tg, HSP60, HMGB1, MHC-II	Dendritic cells	TLR2/3 → NF-κB	DC activation; CD4^+^ Th1/Th17 skewing	(23, 24)
HT	T lymphocytes (tissue)	miR-142-3p	Regulatory T cells	RAC1/ERK1/2 ↓	Treg dysfunction; thyrocyte destruction	(8)
HT	Thyrocytes (IFN-γ stimulated)	TPO, HSP60, MHC-II, ICAM-1	DCs, CD4^+^ T cells	CD40/CD80/CD83 ↑	Pro-inflammatory DC polarization; IFN-γ/IL-17A/IL-22 release	(24)
GD	Circulating exosomes	IGF-1R, HSP60	PBMCs	MyD88/TRIF/p-P65	IL-6, IL-1β secretion	(33)
T1DM	Pancreatic β-cells	GAD65, IA-2, proinsulin, PD-L1	DCs, macrophages, CD8^+^ T cells	Antigen presentation; PD-1 engagement	Autoreactive T-cell priming; biomarker of disease evolution	(13, 28, 31, 34)
T1DM	Stressed β-cells	miR-21-5p, CXCL10	Monocytes, β-cells (autocrine)	CXCL10/CXCR3	Islet inflammation; β-cell dysfunction	(35–37)
T1DM	T lymphocytes	miR-142-3p, miR-142-5p, miR-155	Pancreatic β-cells	Ccl2/Ccl7/Cxcl10 induction	β-cell apoptosis	(38)
T2DM	Adipose tissue macrophages	miR-155	Liver, skeletal muscle	PPARγ ↓	Insulin resistance; glucose intolerance	(39)
T2DM	Adipocytes	miR-34a	Macrophages	Klf4 ↓	Suppressed M2 polarization; adipose inflammation	(40)
T2DM	Visceral adipocytes	miR-155	Skeletal muscle	MyoD ↓	Muscle dysplasia in obesity	(41)
T2DM	M1 macrophages (acute pancreatitis)	Inflammatory mitochondria	β-cells	Ferroptosis pathway	Cross-organ inflammatory–metabolic injury	(32)

↑, increase/upregulation; ↓, decrease/downregulation; →, directional effect, association, transition, or transfer. DC, dendritic cell; GAD65, glutamic acid decarboxylase 65; GD, Graves’ disease; HMGB1, high-mobility group box 1; HSP60, heat shock protein 60; HT, Hashimoto’s thyroiditis; IA-2, islet antigen 2; ICAM-1, intercellular adhesion molecule 1; IGF-1R, insulin-like growth factor 1 receptor; MHC-II, major histocompatibility complex class II; PBMCs, peripheral blood mononuclear cells; PD-L1, programmed death-ligand 1; T1DM, type 1 diabetes mellitus; T2DM, type 2 diabetes mellitus; Tg, thyroglobulin; TPO, thyroid peroxidase.

### 2.5 Shared recipient side: DCs, tissue-resident macrophages and Treg cells

The biological effects of EVs on the recipient side are jointly determined by three variables: which cell internalizes the EV, where the cargo is released, and which pathway is activated. Within the HT–T2DM comparative framework, three categories of recipient cells constitute the shared response hub.

DCs are the principal recipient cells for circulating EVs. In HT, circulating exosomes activate DCs and induce imbalanced antigen presentation (24). MHC-II molecules play a central role in antigen presentation in autoimmune diseases, and the mechanism by which DCs drive imbalanced CD4^+^ T-cell differentiation through MHC-II represents a cross-disease commonality (42).

Tissue-resident macrophages in the pancreatic islets and the thyroid share similar transcriptomic features. Resident macrophages in both tissues preferentially adopt a “sentinel-like” subset profile characterized by self-renewal, antigen clearance and tissue repair (11). This commonality partly explains why the two tissues exhibit convergent responses to circulating EV signals.

At the Treg level, both diseases display reduced Treg numbers and decreased FOXP3 stability (12). Direct targeting of Treg cells by EV-miRNAs is a shared mechanism of functional Treg attenuation in the two conditions (8).

EV tissue tropism is determined by surface integrins and accessory molecules. These surface molecules enable directional enrichment of circulating EVs in distant organs through integrin–extracellular matrix (integrin–ECM) pairing (43). The thyroid and pancreatic islets share fenestrated capillary architecture and high basal metabolic activity, which together provide the anatomical basis for simultaneous accumulation of circulating EVs in both tissues.

### 2.6 Section synthesis: from comparative framework to cross-disease mechanism

The evidence boundary introduced in §2.0 should be carried through the comparative framework that follows.

EVs released from thyroid, pancreatic islets, adipose tissue, liver, gut and bone share overlapping inflammatory cargo and convergent miRNA programs, supporting a cross-disease network of thyro-metaflammation (**Figure 1**). This framework links HT and T2DM while preserving the mechanistic boundary between T1DM and classical T2DM.

## 3 Immune microenvironment dysregulation: the shared pathological baseline of Hashimoto’s thyroiditis and diabetes mellitus

### 3.1 The immune microenvironment as a regulated network

The immune microenvironment is a spatially organized regulatory network composed of immune cells, stromal cells, soluble mediators, and extracellular matrix components, in which T and B lymphocytes, macrophages, DCs, and natural killer cells communicate through cytokines and chemokines (10, 44). Under homeostatic conditions, this network sustains tolerance to self-antigens largely through the suppressive function of FOXP3^+^ Tregs (12). When genetic susceptibility intersects with environmental triggers, the equilibrium shifts from a regulatory- to a pro-inflammatory–dominant state, culminating in organ-specific autoimmune injury (10). High-resolution technologies—spatial transcriptomics and single-cell RNA sequencing—have begun to dissect this landscape with unprecedented granularity (21, 45, 46), revealing that HT, T1DM, and T2DM share strikingly convergent patterns of microenvironmental disturbance. The following sections distill these patterns into four interlocking mechanistic axes that together constitute the immune baseline upon which EV-mediated signaling operates.

### 3.2 Four shared axes of immune microenvironment dysregulation

#### 3.2.1 Treg/Th17 imbalance: the central failure node

Treg numerical decline and functional impairment constitute a core immunological signature shared by HT, T1DM, and T2DM (12). In HT, peripheral Treg frequency is reduced, the Th17/Treg ratio is elevated in proportion to disease activity (47), and aberrant IL-17/Th17 axis activation persists throughout disease progression (48); Mendelian randomization analyses further support a causal relationship between specific T-cell subset signatures and HT risk (49). In T1DM, Treg dysfunction is tightly linked to defective IL-2 signaling (44), while in T2DM islet-local Treg/Th17 imbalance reinforces chronic low-grade inflammation and insulin resistance (11). Across all three conditions, Tregs lose not only quantitative representation but also FOXP3 stability and suppressive plasticity (12). Critically, this functional collapse is not an isolated event: T-lymphocyte–derived small EVs deliver miR-142-3p directly to Tregs, executing the molecular step that translates microenvironmental imbalance into Treg failure (8)—a link that anchors the EV mechanisms developed in Section 4.

#### 3.2.2 Effector T-cell hyperactivation and target-cell destruction

Effector T-cell expansion provides the cytotoxic execution arm of the inflammatory shift. In HT thyroid tissue, Th1 cells predominate, and IFN-γ induces aberrant MHC class II expression on follicular cells, converting them into facultative antigen-presenting targets of their own immune attack (10). In T1DM, autoreactive CD8^+^ T cells recognize preproinsulin, GAD65, IA-2, and ZnT8 and destroy β cells through perforin/granzyme- and Fas/FasL-mediated cytotoxicity (50); single-cell transcriptomics has resolved the heterogeneity of pancreas-infiltrating CD8^+^ T cells and their disease-stage dynamics (45), and the CXCL12–CXCR4 axis amplifies CD57^+^ CD8^+^ effector-memory expansion to accelerate disease progression (51). T follicular helper (Tfh) and T peripheral helper (Tph) populations are likewise expanded in AITD and support autoreactive B-cell responses through IL-21 (52). The pro-inflammatory cytokine network connecting these effector compartments—IL-1β, TNF-α, IFN-γ, and the CXCR3 ligands CXCL9/10/11—is elevated across HT, Graves’ disease, and T1DM, indicating a shared Th1-skewed inflammatory backbone (53, 54), while NLRP3 inflammasome activation amplifies IL-1β and IL-18 release that perpetuates β-cell autoimmune destruction (55). EV cargo enriched in these very chemokines, particularly CXCL10 within β-cell EVs (Section 5), provides the molecular conduit through which this cytokine axis is propagated beyond cell-to-cell contact (37).

#### 3.2.3 Macrophage M1/M2 polarization imbalance and innate immune activation

Dysregulated M1/M2 macrophage balance constitutes the innate-immune pillar of microenvironmental disturbance. In T1DM islets, M1-skewed polarization underlies insulitis and β-cell dysfunction, and pharmacological reprogramming with 4-octyl itaconate attenuates both insulitis and metabolic deterioration in non-obese diabetic (NOD) mice (11, 56). In HT thyroid tissue, inflammatory macrophages and DCs infiltrate alongside lymphocytes (10), while tolerogenic DC deficiency further compromises peripheral tolerance and represents a tractable cell-based therapeutic target in T1DM (57). In T2DM, M1-polarized adipose tissue macrophages extend this pattern into the systemic compartment, releasing EV-borne miR-155 that imposes insulin resistance on liver and skeletal muscle (39). Thus, M1/M2 imbalance is not confined to the target organ but operates as an inter-organ relay—a function executed largely through EV-mediated miRNA transfer (Sections 5–6).

#### 3.2.4 Aberrant B-cell responses and humoral immune dysregulation

B-cell–driven humoral immunity completes the shared dysregulation pattern. In HT, B-cell and plasma-cell infiltration of thyroid tissue sustains anti-thyroid peroxidase and anti-thyroglobulin autoantibody production (10), and single-cell atlases have identified disease-associated B-cell subsets across thyroid tissue and peripheral blood (46). In T1DM, islet antigen–reactive B cells activate autoreactive T cells through antigen presentation and secrete autoantibodies against GAD65, IA-2, and ZnT8 (58). The mechanistic link to EV biology is direct: in T1DM, β-cell–derived EVs carry these same islet autoantigens and pro-inflammatory signals into the islet microenvironment, where they function as mobile antigen-presenting units that prime both B- and T-cell autoreactivity (13); this humoral–EV coupling is a T1DM-specific mechanism and is not extended to classical T2DM, where B-cell/humoral autoreactivity is not a defining feature. This positions B-cell humoral output and EV-borne autoantigen delivery as two reinforcing arms of the same tolerance-breakdown loop in HT and T1DM.

### 3.3 From microenvironmental baseline to EV-mediated signaling

The four axes outlined above—Treg/Th17 collapse, effector T-cell hyperactivation, M1-skewed macrophage polarization, and aberrant B-cell humoral output—define a stereotyped pro-inflammatory baseline shared by HT, T1DM, and T2DM (with the caveat, per §2.0 and §3.2.4, that B-cell/humoral autoreactivity applies to HT and T1DM rather than classical T2DM). Yet this microenvironmental description leaves a central question unanswered: by what molecular medium are these dysregulated states initiated, propagated between cells, and disseminated across organ boundaries? Classical cytokine and chemokine networks account for short-range diffusible signaling, but they cannot explain how autoantigens, intracellular regulatory miRNAs, and DAMPs are transferred intact between distant cells, nor how thyroid and pancreatic compartments come to share overlapping immune signatures in comorbid patients.

Extracellular vesicles fill precisely this explanatory gap. EVs simultaneously carry MHC–peptide complexes, intracellular autoantigens, immunoregulatory miRNAs, cytokines, and DAMPs (6, 7), enabling them to act as integrated information packets that transmit each of the four dysregulation axes between donor and recipient cells. T-cell–derived EVs execute Treg failure through miR-142-3p delivery (8); β-cell EVs propagate cytokine-axis activation through CXCL10 enrichment (37); adipose macrophage EVs disseminate M1 polarization signals through miR-155 transfer (39); and, in T1DM specifically, β-cell EVs convey islet autoantigens to professional antigen-presenting cells, sustaining humoral autoreactivity (13). Microenvironmental dysregulation and EV-mediated signaling are therefore not parallel phenomena but two descriptive levels of the same pathological process—the cellular phenotype and its molecular medium.

The following sections focus on how EVs transmit these shared immune-microenvironment disturbances in HT and diabetes.

## 4 The role of extracellular vesicles in Hashimoto’s thyroiditis

### 4.1 Origin and HT-specific characteristics of EVs in HT

Hashimoto’s thyroiditis (HT) is the most common organ-specific autoimmune thyroid disease. It is characterized by diffuse lymphocytic infiltration, persistently elevated anti-thyroid antibodies (TPOAb and TgAb), and progressive destruction of thyroid follicles (10). The global incidence of HT continues to rise, and HT is now the leading cause of adult hypothyroidism (1). Clinically, HT may present as a diffuse goiter or an atrophic thyroid, and a subset of patients have coexisting autoimmune endocrine disorders (59).

**Figure 2:**
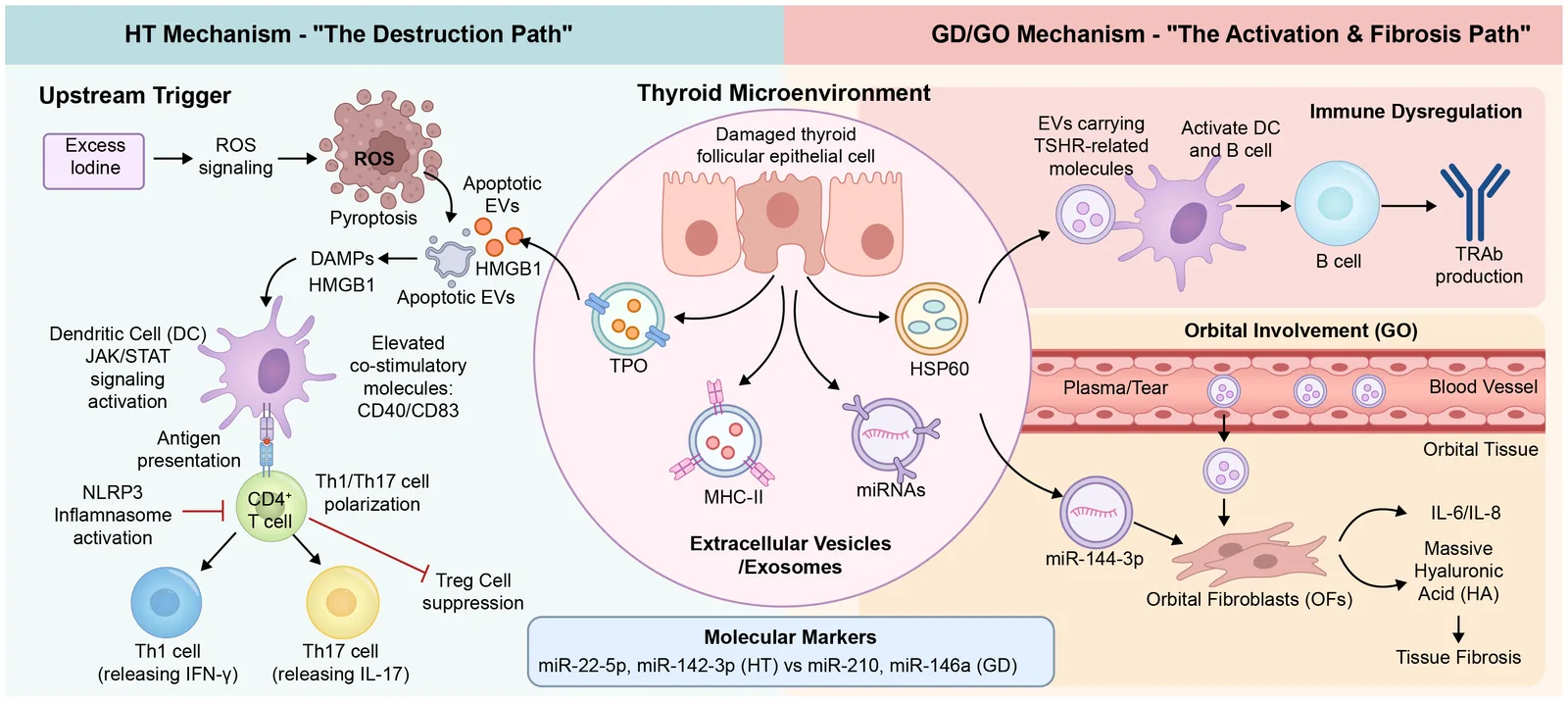
Divergent EV-mediated pathological circuits in autoimmune thyroid disease: the HT “destruction path” versus the GD/GO “activation and fibrosis path”. Left panel (HT mechanism): Excess iodine exposure triggers ROS signaling and pyroptosis in thyroid follicular epithelial cells, releasing apoptotic EVs enriched in HMGB1 and other DAMPs. These EVs activate dendritic cells via JAK/STAT signaling, upregulate CD40 and CD83 co-stimulatory molecules, and drive antigen presentation to CD4^+^ T cells. NLRP3 inflammasome activation reinforces Th1/Th17 polarization (IFN-γ and IL-17 release) while suppressing Treg function, culminating in follicular destruction. Center panel (Thyroid microenvironment): Damaged follicular cells release EVs/exosomes carrying TPO, MHC-II, HSP60 and immunoregulatory miRNAs, which serve as the shared molecular hub. Right panel (GD/GO mechanism): EVs bearing TSHR-related molecules activate the DC–B-cell axis to drive TRAb production; plasma- and tear-derived EVs carrying miR-144-3p reach orbital fibroblasts through the orbital vasculature, stimulating IL-6 and IL-8 secretion and massive hyaluronic acid production, ultimately causing tissue fibrosis in Graves’ ophthalmopathy. Discriminating circulating miRNA signatures: miR-22-5p/miR-142-3p (HT) versus miR-210/miR-146a (GD). CD4^+^, cluster of differentiation 4 positive T cell; CD40/CD83, co-stimulatory molecules on dendritic cells; DAMPs, damage-associated molecular patterns; DC, dendritic cell; EVs, extracellular vesicles; GD, Graves’ disease; GO, Graves’ ophthalmopathy; HA, hyaluronic acid; HMGB1, high-mobility group box 1; HSP60, heat shock protein 60; HT, Hashimoto’s thyroiditis; IFN-γ, interferon gamma; IL-6/IL-8/IL-17, interleukin 6/8/17; JAK/STAT, Janus kinase/signal transducer and activator of transcription; MHC-II, major histocompatibility complex class II; miRNA, microRNA; NLRP3, NLR-family pyrin domain containing 3; OFs, orbital fibroblasts; ROS, reactive oxygen species; Th1/Th17, T-helper 1/17 cell; TPO, thyroid peroxidase; TRAb, thyrotropin receptor antibody; Treg, regulatory T cell; TSHR, thyrotropin (thyroid-stimulating hormone) receptor.

What is HT-specific in the EV literature. To make the HT-specific angle of this review explicit, the following observations are consolidated here rather than distributed across later sections. First, HT-derived EVs carry a defining triad of thyroid autoantigens—TPO, Tg and HSP60—together with MHC class II molecules; this cargo signature is not shared with the diabetes EV literature and constitutes the direct molecular substrate for HT-specific autoantigen presentation (23). Second, T-lymphocyte–derived tissue small EVs in HT thyroid tissue are enriched in miR-142-3p, and this cargo has been functionally shown to impair Treg suppressive function and to accelerate thyrocyte destruction (8)—one of the very few HT-side EV cargoes with functional cargo-transfer evidence. Third, EV cargo signatures diverge sharply between HT and Graves’ disease/Graves’ ophthalmopathy (GD/GO) despite their shared AITD origin: HT-derived EVs preferentially route through a DAMP–DC–Th1/Th17 “destruction path,” whereas GD/GO-related EVs engage a TSHR/IGF-1R “activation-and-fibrosis path” (see §4.3; **Figure 2**). Where subsequent HT-side inferences depend on extrapolation from T2DM-side evidence rather than on direct HT data, this dependence is flagged explicitly, per the evidence-asymmetry caveat introduced in §2.0.

In HT, EVs originate predominantly from thyroid follicular epithelial cells, infiltrating T and B lymphocytes, and DCs (60). Under pathological conditions, thyrocytes release EVs with altered miRNA cargo that contribute to immune microenvironment remodeling (14). Serum exosomes from HT patients highly express TPO, HSP60, and MHC-II molecules; they are internalized by DCs and trigger CD4^+^ T-cell polarization imbalance via the TLR2/3–NF-κB pathway (23). IFN-γ–stimulated thyrocyte-derived exosomes carry TPO, HSP60, and MHC-II, function as antigen-presenting cargo, and drive CD4^+^ T cells to secrete pro-inflammatory cytokines including IFN-γ, IL-17A, and IL-22 (24).

At the tissue level, T-lymphocyte–derived small EVs in HT thyroid tissue are enriched in miR-142-3p, which impairs Treg suppressive function upon uptake and promotes thyrocyte apoptosis (8). Differential miRNA profiles have been identified in circulating exosomes of HT and Graves’ disease patients, mapping to key immunoregulatory pathways (61). Proteomic analysis of circulating EVs in AITD shows significant upregulation of proteins involved in complement activation, antigen presentation, and inflammatory signaling (27).

### 4.2 EVs modulate T- and B-cell immune responses

The core immune mechanism of HT involves enhanced Th1/Th17 responses with impaired Treg/Th2 function, and EVs serve as pivotal regulators in this process (62). As intercellular signaling carriers, EVs deliver proteins, miRNAs, and metabolites that shape inflammatory and immune responses (16).

Exosomes derived from DCs and thyrocytes carry MHC–peptide complexes and costimulatory molecules that activate naive CD4^+^ T cells (63). Within the HT microenvironment, IFN-γ–induced thyrocyte exosomes upregulate CD40, CD80, and CD83 on DCs and drive them toward a pro-inflammatory phenotype (24). MHC-II presentation plays a central role in regulating autoreactive T-cell function (42).

Th17-associated molecular programs can be amplified and sustained by small EVs (64). Aberrant PD-L1 signaling within the HT thyroid microenvironment contributes to defective T-cell suppression (65). B-cell– and plasma-cell–derived exosomes carry BCR components and immunoglobulin fragments that participate in autoantibody formation and dissemination (66).

Let-7 family miRNAs negatively regulate Th17 differentiation, suggesting that EV-encapsulated miRNAs may participate in maintaining immune tolerance (67).

### 4.3 EVs mediate thyrocyte injury and amplification of inflammation

In HT, thyroid tissue destruction depends not only on CD8^+^ T-cell cytotoxicity and antibody-dependent cellular cytotoxicity (ADCC) but also on EV-mediated chronic inflammatory amplification and sustained epithelial injury (22). The apoptotic pathways involved in HT thyrocyte destruction include Fas/FasL, TNF-α/TNFR1, and perforin/granzyme B (10).

Within the HT thyroid microenvironment, sustained IFN-γ and TNF-α exposure induces thyrocytes to release pro-inflammatory exosomes containing HSP60, HMGB1, and ICAM-1 (23). Complex regulatory networks among Th1 cells, Th17 cells, and activated B cells within HT tissue constitute a critical node of immune dysregulation (30).

In peripheral blood, the circulating exosomal miRNA profile of HT patients is altered and is associated with activation of inflammatory immune pathways (68). Pro-inflammatory miRNAs within peripheral EVs correlate with the severity of hypothyroidism in HT (69).

The mechanistic divergence between HT and Graves’ disease/Graves’ ophthalmopathy (GD/GO)—despite their shared autoimmune origin—is largely encoded by EV cargo specificity (**Figure 2**). HT-derived EVs follow a “destruction path” centered on DAMP–DC–Th1/Th17 amplification, whereas GD/GO-related EVs follow an “activation and fibrosis path” that engages TSHR-reactive B cells and orbital fibroblasts. This dual-trajectory model rationalizes why distinct circulating miRNA signatures (miR-22-5p/miR-142-3p versus miR-210/miR-146a) can discriminate between the two clinical phenotypes. The corresponding pathway-evidence citations supporting the HT (23, 27, 68) and GD/GO (33, 61) arms of **Figure 2** are provided in the figure caption, and the caption now states that the figure is an original schematic drawn by our team using Adobe Illustrator, with no reuse of third-party pathway diagrams.

### 4.4 EVs as potential biomarkers for HT

EVs are stably detectable in peripheral blood and thyroid tissue, and their disease-specific composition makes them attractive non-invasive biomarkers for HT (70). The MIBlood-EV reporting framework provides standardized recommendations for sample collection, EV isolation, and data reporting in blood-derived EV studies (70).

At the protein level, thyrocyte-derived exosomes carrying HSP60, TPO and MHC-II have been shown *in vitro* to activate DCs through TLR2/3–NF-κB signaling and to induce imbalanced CD4^+^ T-cell responses (23). This mechanistic evidence is derived from thyroid cell-line and DC coculture systems. Inflammation- and adhesion-related proteins, including complement components, ICAM-1, and CD44, are markedly upregulated in serum EVs of patients with AITD (27).

At the miRNA level, circulating EV miRNAs have diagnostic potential in HT, and miR-142-3p has been validated in large cohorts (8). EV-derived miRNAs may also serve as indicators of disease activity in autoimmune thyroid disorders (71).

Further progress in HT biomarker development will require MISEV2023-compliant standardization and multicenter clinical validation (6). Candidate HT-related EV biomarkers are summarized in **Table 2**.

**Table 2:** Candidate EV-based biomarkers for HT, T1DM, and T2DM.

Disease	Biomarker	Specimen	Direction	Clinical correlation	Reference
HT	HSP60, TPO, Tg, MHC-II (serum sEV proteins)	Serum sEV	↑	Correlated with TPOAb/TgAb titers	(23)
HT	Complement components, ICAM-1, CD44	Serum EV	↑	Inflammatory and adhesion activation	(27)
HT	miR-142-3p (tissue/circulating)	Thyroid + serum	↑	Validated in large cohort	(8)
HT/AITD	Circulating exosomal miRNA panel	Plasma	Differential	Disease activity indicator	(68, 71)
HT	Peripheral EV pro-inflammatory miRNAs	Plasma EV	↑	Severity of hypothyroidism	(69)
HT vs GD	miR-15a-5p, miR-126-3p, miR-142-5p, miR-21-5p, miR-150-5p	Circulating	Differential	Discriminates GD vs HT; correlates with thyroid hormone/antibodies	(72)
T1DM	β-cell EV PD-L1	Plasma	↑ in high-risk	Correlates with C-peptide; disease evolution biomarker	(31)
T1DM	EV miR-21-5p	Serum (NOD mice + children)	↑ 3–6 fold	T1D development biomarker	(36)
T1DM (long duration)	Plasma EV miRNA signature	Plasma	Distinct signature	Immune activation; β-cell stress	(73)
T2DM + IHD	Six-miRNA EV panel (miR-15a-3p/18a-5p/133a-3p/155-5p/210-3p/19a-3p)	Circulating EV	Differential	Distinguishes patients from controls	(74)
T2DM	EV miR-20b-5p	Circulating exosomes	↑	Skeletal-muscle insulin resistance	(75)
Diabetic dyslipidemia	miR-218, miR-132, miR-143, miR-21, miR-122, miR-155	Plasma + microvesicles	Differential	Diagnostic and prognostic value	(76)
Prediabetes	EV miRNA signature	Circulating EV	Altered	Pre-clinical high-risk identification	(77)
DKD	Urinary/blood exosomal miRNAs	Urinary EV	Differential	Outperforms microalbuminuria	(78)

↑, upregulated. AITD, autoimmune thyroid disease; DKD, diabetic kidney disease; EV, extracellular vesicle; GD, Graves’ disease; HSP60, heat shock protein 60; HT, Hashimoto’s thyroiditis; ICAM-1, intercellular adhesion molecule 1; IHD, ischemic heart disease; MHC-II, major histocompatibility complex class II; NOD, non-obese diabetic; PD-L1, programmed death-ligand 1; sEV, small extracellular vesicle; T1D/T1DM, type 1 diabetes/type 1 diabetes mellitus; T2DM, type 2 diabetes mellitus; Tg, thyroglobulin; TgAb, anti-thyroglobulin antibody; TPO, thyroid peroxidase; TPOAb, anti-thyroid peroxidase antibody.

## 5 The role of extracellular vesicles in diabetes mellitus

### 5.1 Type 1 diabetes: extracellular vesicles and autoimmune attack on β-cells

T1DM is characterized pathologically by progressive autoimmune destruction of pancreatic β-cells, with disease onset arising from a complex interaction among genetic susceptibility, environmental triggers, and dysregulated immunity. EVs have emerged as key mediators of intercellular communication within the islet microenvironment and play a central role in the initiation and amplification of autoimmunity in T1DM (13).

Under physiological stress or cytokine stimulation, β-cells release EVs loaded with core T1DM autoantigens, including proinsulin, GAD65, and IA-2 (34). These β-cell–derived EVs enter the islet microenvironment, where they are efficiently taken up by dendritic cells and macrophages and presented to T cells, thereby activating autoreactive T cells that mount targeted immune attacks on β-cells (28). β-cells exposed to inflammatory, hypoxic, or genotoxic stress release distinct EV subpopulations whose abundance, molecular composition, and immunogenicity are markedly altered (79). EVs released from stressed β-cells directly activate peripheral blood monocytes, contributing to the establishment of local islet inflammation (35).

β-cell–derived EVs also exert paracrine injury on neighboring cells through specific miRNA cargo. Exposure to inflammatory cytokines (IL-1β, IFN-γ, TNF-α) increases the miR-21-5p content of β-cell–derived EVs by three- to six-fold; this elevation has been confirmed in serum EVs from NOD mice and from children with newly diagnosed T1DM, supporting its value as a circulating biomarker of T1DM development (36). Conversely, EVs derived from immune cells (T cells, macrophages, and B cells) deliver specific miRNAs that promote β-cell apoptosis and contribute to T1DM progression (80).

EV-carried miRNAs can also drive antigen-independent “bystander activation” by mimicking pathogen-derived single-stranded RNA and engaging endosomal TLR7/8 signaling (81). Upon pro-inflammatory cytokine stimulation, β-cell EVs become enriched with the chemokine CXCL10, which engages CXCR3 to impair β-cell function and recruit immune cells into the islets (37). Circulating EV PD-L1 levels are elevated in individuals at high risk of T1DM and correlate with residual C-peptide, suggesting that β-cell EV PD-L1 may serve as a biomarker reflecting β-cell stress and immune modulation (31). In addition, the senescence-associated secretory phenotype (SASP) of aging β-cells modifies EV release and cargo composition, further aggravating autoantigen exposure and immune signaling (82).

### 5.2 Type 2 diabetes: extracellular vesicles in chronic inflammation and insulin resistance

The core pathophysiology of T2DM consists of insulin resistance and progressive β-cell failure, both of which are tightly linked to low-grade chronic inflammation originating in adipose tissue. Consistent with the mechanistic boundary established in §2.3.1, T2DM is treated here as a metabolic-inflammatory disease in which EVs act as carriers of stress signals, chemokines and immunoregulatory miRNAs, rather than as an autoantigen-driven platform; the autoimmune subgroup LADA is discussed separately from classical T2DM. miRNAs delivered by adipose tissue–derived EVs serve as key molecular mediators linking obesity, inflammation, and target-organ insulin resistance (83).

In obesity, EVs secreted by adipose tissue macrophages (ATMs) are enriched in miR-155 and can be taken up by insulin target organs such as liver and skeletal muscle, where they target PPARγ and induce insulin resistance and glucose intolerance; mice deficient in miR-155 are protected against high-fat-diet–induced insulin resistance (39). Adipocyte-secreted exosomes carrying miR-34a suppress M2 macrophage polarization through targeting Klf4, thereby aggravating adipose-tissue inflammation and metabolic dysfunction (40).

Adipocytes can also release lipid-rich vesicles (“AdExos”) via a lipase-independent pathway; these vesicles serve as a local source of lipids for macrophages and participate in obesity-associated immune regulation (84). At the level of skeletal muscle, visceral adipocyte-derived EVs deliver miR-155, which directly suppresses the myogenic differentiation marker MyoD and disrupts muscle homeostasis (41).

ATM-derived EVs also act on pancreatic β-cells. EVs from obese mouse ATMs are enriched in miRNAs that modulate β-cell function, and this effect is markedly attenuated when ATM EVs are derived from Dicer-knockout mice (85). Collectively, EV-mediated “adipose–immune–metabolic” crosstalk establishes a multi-organ chronic inflammatory network that underlies T2DM (86).

**Figure 3:**
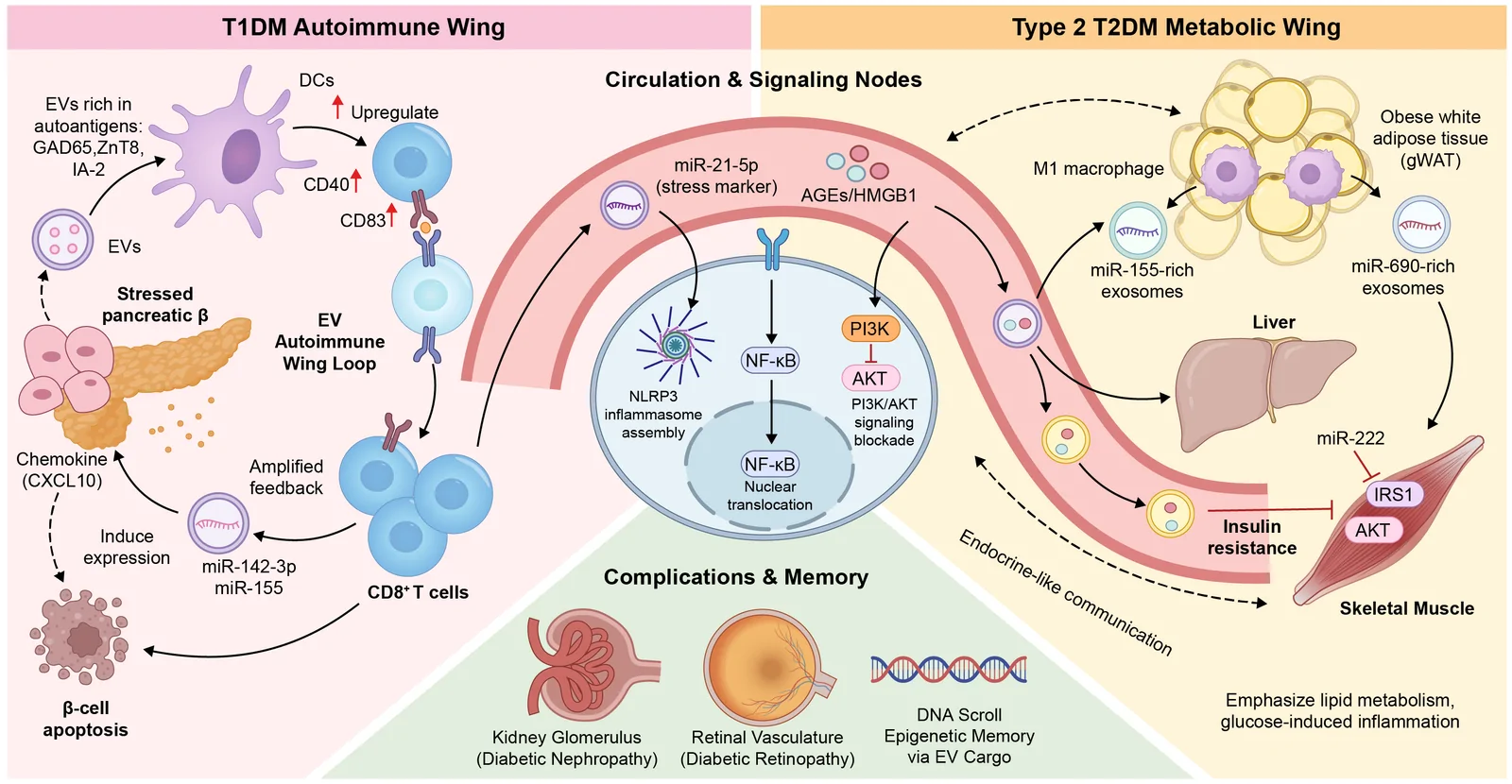
The “dual-wing” model of EV-driven pathogenesis in diabetes mellitus. Left wing (T1DM autoimmune arm): Stressed pancreatic β-cells release EVs enriched in islet autoantigens (GAD65, ZnT8, IA-2). These EVs activate dendritic cells with upregulated CD40 and CD83 expression, prime CD8^+^ T cells, and amplify the autoimmune feedback loop through T-cell–derived miR-142-3p and miR-155, which induce chemokine CXCL10 expression and trigger β-cell apoptosis. Central node (Circulation and signaling): Circulating EVs carrying miR-21-5p (stress marker) and AGEs/HMGB1 converge on recipient cells, activating the NLRP3 inflammasome and NF-κB nuclear translocation while suppressing PI3K/AKT–mediated insulin signaling. Right wing (T2DM metabolic arm): Obese white adipose tissue M1 macrophages release miR-155-rich and miR-690-rich exosomes that act on liver and skeletal muscle in an endocrine-like manner; miR-222 in particular suppresses IRS1/AKT signaling, producing systemic insulin resistance. Bottom panel (Complications and memory): EV cargo participates in diabetic nephropathy, diabetic retinopathy, and the establishment of epigenetic “metabolic memory” through DNA-modifying signals. AGEs, advanced glycation end-products; AKT, protein kinase B; CD8^+^, cluster of differentiation 8 positive T cell; CD40/CD83, co-stimulatory molecules on dendritic cells; CXCL10, C-X-C motif chemokine ligand 10; DCs, dendritic cells; EVs, extracellular vesicles; GAD65, glutamic acid decarboxylase 65; gWAT, gonadal (visceral) white adipose tissue; HMGB1, high-mobility group box 1; IA-2, islet antigen 2; IRS1, insulin receptor substrate 1; M1, classically activated (pro-inflammatory) macrophage phenotype; miRNA, microRNA; NF-κB, nuclear factor kappa B; NLRP3, NLR-family pyrin domain containing 3; PI3K, phosphoinositide 3-kinase; T1DM, type 1 diabetes mellitus; T2DM, type 2 diabetes mellitus; ZnT8, zinc transporter 8.

T1DM and T2DM represent two mechanistically distinct wings of the EV network in diabetes (**Figure 3**): T1DM is dominated by β-cell autoantigen transfer, whereas T2DM is dominated by metabolic-inflammatory EV signaling; their convergence lies mainly in downstream inflammatory and insulin-signaling pathways rather than shared cargo identity. The cell-source–resolved evidence across diabetic complications is summarized in **Table 3**.

**Table 3:** Cell-source-resolved evidence for EV involvement in diabetic complications.

Complication	Donor cell	EV cargo	Target cell	Mechanism	Reference
Atherosclerosis/prothrombosis	Platelets (T2D)	—	Vascular wall	PAR-4/Ca²^+^-calpain activation → PDMP formation	(87)
Endothelial injury	HUVECs (high glucose)	—	Vascular endothelium	Pro-oxidative endothelial microparticles	(88)
Vascular damage	Monocytes (high glucose)	miR-142-5p	Endothelial cells	Impaired endothelial function	(89)
Vascular repair deficit	Circulating EV	miR-126 ↓	Vascular cells	Loss of vascular integrity signaling	(90)
DKD — podocyte injury	Glomerular endothelial cells	TGF-β1 mRNA	Podocytes	EMT; Wnt/β-catenin activation	(91)
DKD — podocyte apoptosis	Urinary exosomes	miR-145-5p	Podocytes	Srgap2 ↓; RhoA/ROCK ↑	(92)
DKD — diagnostic markers	Urinary exosomes	miR-21-5p ↑/miR-30b-5p ↓	—	Differential expression	(93)
DKD — fibrosis therapy	Placental MSC-EVs	miR-99b-5p	Renal cells	mTOR/autophagy axis	(94)
DR — proliferative	Plasma EVs	miR-30b	Retinal microvascular endothelium	SIRT1 ↓; VEGF ↑	(95)
DR — pericyte–endothelial crosstalk	Pericytes	circRNA cPWWP2A	Endothelial cells	Sponge of miR-579; Ang-1, occludin, SIRT1 regulation	(96)
DR — neovascularization	Retinal pigment epithelium (oxidative stress)	—	Endothelial cells	Enhanced exosome secretion; angiogenesis	(97)
DPN — myelin repair	Schwann cells	miRNA cargo	Peripheral nerves	Restoration of nerve conduction velocity	(98)
Diabetic wound	M1 macrophages	miR-155-5p	Vascular endothelium	GDF6 ↓; impaired angiogenesis	(99)

↑, increase/upregulation. Ang-1, angiopoietin-1; DKD, diabetic kidney disease; DPN, diabetic peripheral neuropathy; DR, diabetic retinopathy; EMT, epithelial–mesenchymal transition; GDF6, growth differentiation factor 6; HUVECs, human umbilical vein endothelial cells; MSC-EVs, mesenchymal stem cell–derived extracellular vesicles; mTOR, mechanistic target of rapamycin; PAR-4, protease-activated receptor 4; PDMP, platelet-derived microparticle; SIRT1, sirtuin 1; T2D, type 2 diabetes; TGF-β1, transforming growth factor beta 1; VEGF, vascular endothelial growth factor.

### 5.3 Extracellular vesicles in diabetic complications

Chronic diabetic complications include macrovascular disease typified by atherosclerosis, together with microvascular complications such as diabetic kidney disease (DKD), diabetic retinopathy (DR), and diabetic peripheral neuropathy (DPN). The hyperglycemic microenvironment induces aberrant EV secretion from multiple cell types, driving the onset and progression of these complications (100).

#### 5.3.1 Macrovascular disease and atherosclerosis

In patients with T2DM, hyperglycemia activates the PAR-4/Ca²^+^-calpain pathway to promote the formation of platelet-derived microparticles (PDMPs), establishing PDMPs as important markers and mediators of the diabetic prothrombotic state (87). Cultured human umbilical vein endothelial cells exposed to high glucose release greater numbers of endothelial microparticles (EMPs) with enhanced pro-oxidative activity, supporting EMPs as both markers and effectors of vascular injury (88). Monocyte-derived EVs generated under high glucose impair endothelial function through delivery of miR-142-5p, highlighting a role for innate immune cell–endothelial EV communication in diabetic vascular damage (89). In T2DM, reduced EV-associated miR-126 directly compromises vascular repair and integrity and correlates with the development of both microvascular and macrovascular complications (90).

#### 5.3.2 Diabetic kidney disease

EV-mediated intercellular communication plays a central regulatory role in glomerulosclerosis and tubulointerstitial fibrosis in DKD. Under high-glucose conditions, glomerular endothelial cells release exosomes enriched in TGF-β1 mRNA; uptake of these exosomes by adjacent podocytes induces epithelial–mesenchymal transition and activates Wnt/β-catenin signaling, aggravating glomerular filtration barrier injury (91). Urinary exosomal miR-145-5p is markedly upregulated in DKD patients and induces podocyte apoptosis via suppression of Srgap2 and activation of the RhoA/ROCK pathway (92). Multicenter studies confirm that urinary exosomal miR-21-5p is elevated and miR-30b-5p is reduced in DKD, supporting both as candidate diagnostic biomarkers (93). From a therapeutic perspective, EVs from human placental mesenchymal stem cells deliver miR-99b-5p to attenuate renal fibrosis through the mTOR/autophagy axis, demonstrating the dual diagnostic and therapeutic value of EVs in DKD (94).

#### 5.3.3 Diabetic retinopathy

DR is characterized by retinal microvascular leakage, neovascularization, and neurodegeneration. EVs from multiple cellular sources show marked changes in secretion under hyperglycemic conditions and are closely linked to DR progression (101). Plasma-derived EVs carrying miR-30b target SIRT1 to upregulate VEGF and enhance high-glucose–induced angiogenesis in retinal microvascular endothelial cells, contributing to the development of proliferative DR (95). Circulating EVs are significantly increased in diabetic patients with retinopathy and can induce pericyte detachment and migration as well as new vessel formation (102). Within the pericyte–endothelial cell unit, hyperglycemia upregulates the circular RNA cPWWP2A in pericytes; cPWWP2A is then transferred to endothelial cells via exosomes, where it acts as a molecular sponge for miR-579 to regulate Angiopoietin-1, occludin, and SIRT1 expression, ultimately driving microvascular dysfunction (96). In retinal pigment epithelial cells, oxidative stress markedly increases exosome secretion and promotes angiogenesis in recipient endothelial cells, contributing to neovascularization in advanced DR (97).

#### 5.3.4 Diabetic peripheral neuropathy

Schwann cell–derived EVs regulate peripheral nerve myelination and axonal regeneration through their miRNA cargo. Conditional Dicer deletion in Schwann cells causes severe peripheral neurodegeneration, whereas EVs from healthy Schwann cells effectively restore nerve conduction velocity and promote myelin repair in diabetic mice via miRNA delivery (98).

### 5.4 Extracellular vesicles as diagnostic and monitoring biomarkers in diabetes

Conventional diagnostic indicators for diabetes—blood glucose, glycated hemoglobin (HbA1c), and islet autoantibodies—typically become abnormal only after substantial tissue damage has occurred. Circulating EVs reflect real-time cellular status, derive from diverse cell types, and carry stable cargo molecules, positioning them as a novel liquid-biopsy platform for early diagnosis and dynamic monitoring of diabetes (103).

#### 5.4.1 EV-carried miRNAs as diagnostic and monitoring indicators

EV-encapsulated miRNAs have attracted attention because of their superior stability in blood relative to free miRNAs. In T2DM patients with ischemic heart disease, a panel of six circulating EV miRNAs (miR-15a-3p, miR-18a-5p, miR-133a-3p, miR-155-5p, miR-210-3p, and miR-19a-3p) effectively distinguishes patients from healthy controls (74). Plasma EVs from individuals with long-standing T1DM display a distinct miRNA signature that may reflect immune activation and β-cell stress (73). In diabetic dyslipidemia, alterations in plasma and microvesicle levels of miR-218, miR-132, miR-143, miR-21, miR-122, and miR-155 provide diagnostic and prognostic information for this subgroup (76).

EV miRNA profiles are already altered during prediabetes, enabling identification of high-risk individuals years before clinical onset of overt diabetes (77). Circulating exosomal miR-20b-5p is elevated in T2DM and targets AKTIP to impair insulin signaling in skeletal muscle, supporting it as a biomarker of T2DM-associated insulin resistance (75).

#### 5.4.2 Urinary EVs in early detection of DKD

Compared with plasma EVs, urinary EVs originate directly from cells along the nephron and therefore offer greater organ specificity for DKD. A systematic review and meta-analysis show that multiple miRNAs in urinary and blood-derived exosomes are significantly altered in DKD patients relative to healthy controls and T2DM patients without nephropathy, with diagnostic performance superior to traditional microalbuminuria measurements (78).

#### 5.4.3 Standardization and clinical translation of EV research

Standardization remains a key bottleneck for clinical translation of EV research. The MISEV2023 guidelines from the International Society for Extracellular Vesicles (ISEV) provide systematic recommendations on EV nomenclature, isolation, characterization, and reporting, advocating use of the term “extracellular vesicles” based on physical and biochemical properties; widespread implementation will improve reproducibility and accelerate the translation of EV biomarkers in diabetes and its complications from bench to bedside (6). At the same time, most of the EV-biomarker candidates cited above derive from single-center studies with modest sample sizes and heterogeneous EV isolation methods; independent multicenter validation with pre-specified analytical performance criteria, and head-to-head comparison against established biomarkers (HbA1c, urinary albumin-to-creatinine ratio, islet autoantibody titers), will be required before any EV-based readout can be considered clinically actionable. This cautious framing is applied throughout the biomarker discussion. The full spectrum of candidate EV-based biomarkers identified across HT, T1DM and T2DM—together with their specimen type, dysregulation direction, and clinical correlations—is consolidated in **Table 2**.

## 6 Common mechanisms by which extracellular vesicles remodel the immune microenvironment

EVs act as central carriers of intercellular communication and transport a wide range of bioactive cargo, including proteins, nucleic acids, and lipids (6). They exert convergent functions in remodeling the immune microenvironment of HT and diabetes mellitus (7). Dissecting these shared mechanisms is important for understanding how EVs break immune tolerance and amplify tissue-specific inflammation, and it provides a theoretical foundation for cross-disease EV-targeted intervention. Following the boundary principle established in §2.3.1, the autoantigen-presentation and epigenetic-priming mechanisms documented in §6.1 and §6.4 are treated here as HT- and T1DM-specific and are not extended to classical T2DM; T2DM-side discussion is confined to metabolic-stress signaling, low-grade chronic inflammation, and insulin-resistance-associated inflammatory amplification.

### 6.1 Antigen presentation and immune activation

The role of EVs as a “mobile antigen-presenting platform” is documented as an autoimmune-initiating mechanism in HT and T1DM; this mechanism is not claimed here for classical T2DM (see the §6 preamble). EVs carry membrane-bound MHC molecules together with self-antigenic peptides and directly activate CD4^+^ and CD8^+^ T cells (104). This pathway bypasses the classical cell-to-cell antigen-presentation route and provides a molecular basis for the long-distance activation of self-reactive T cells.

In AITD, circulating exosomes carry TPO, Tg, HMGB1, and MHC-II complexes, are efficiently internalized by DCs and monocytes, and thereby activate downstream immune responses (20). In GD, circulating exosomes show elevated expression of IGF-1R and HSP60, engage TLR2/3 on the surface of PBMCs, and drive IL-6 and IL-1β secretion through the MyD88/TRIF/p-P65 pathway (33). Plasma exosome proteomics in AITD further reveals differential expression of complement proteins, immunoglobulin-binding proteins, and a broader spectrum of immunoregulatory proteins (105).

A parallel “mobile antigen-presenting” mechanism operates within the islet microenvironment in T1DM. Pancreatic β-cell EVs carry the core self-antigens proinsulin, GAD65, and IA-2 (13). These EVs are taken up by professional antigen-presenting cells in the pancreatic lymph nodes, where antigenic peptides are subsequently presented to islet-reactive T cells (28). This antigen-presentation cascade is a defining feature of T1DM and is not transposed to classical T2DM in the present review; whether an analogous cascade contributes to latent autoimmune diabetes in adults (LADA) is currently hypothesis-generating and is discussed as such in §2.3.1.

Mitochondria-derived EVs also play a distinct role in initiating autoimmunity. They transport mitochondrial antigens such as cytochrome c, mitochondrial DNA, and cardiolipin, which activate antigen-presenting cells and participate in autoimmune priming (106). In parallel, islet-derived EVs loaded with miR-122-5p, miR-192-5p, and miR-375-3p activate phagocytes and enhance the cytotoxic capacity of T and NK cells (107).

### 6.2 Regulation of immunoregulatory cells

EVs exert bidirectional regulatory effects on regulatory T cells (Tregs). In HT, T lymphocyte–derived tissue small EVs deliver miR-142-3p to induce functional defects in Tregs and aggravate thyrocyte destruction (8). Peripheral blood miR-146a is dysregulated in HT and correlates significantly with the Th17/Treg cytokine imbalance (108).

MSC-EVs, as a next-generation cell-free therapeutic platform, have been shown to promote Treg expansion across multiple autoimmune disease models. In experimental autoimmune encephalomyelitis (EAE), human umbilical cord MSC-derived exosomes upregulate Lag-3 expression on Foxp3^+^CD4^+^ T cells and reinforce their suppressive activity (109). In systemic lupus erythematosus (SLE), engineered Foxp1^high^ MSC exosomes precisely reprogram Tregs through the Foxp1/STAT5/Foxp3 axis (110).

In T1DM models, the secretome of human umbilical cord MSCs—which contains EVs together with other soluble factors—markedly expands the splenic Treg compartment, elevates IL-4, IL-10, and TGF-β, and lowers IL-17 and IFN-γ (111). Such bidirectional regulation highlights the potential of MSC-EVs to restore immune tolerance in T1DM.

EV-mediated regulation of myeloid-derived suppressor cells (MDSCs) constitutes another critical arm of the immune-balance axis. MDSC-derived exosomes inhibit T-cell proliferation and induce Treg differentiation through cargo molecules such as arginase I and iNOS (112). Within the tumor microenvironment, MDSC-EVs substantially suppress T-cell function via multiple EV cargo proteins (113). The MDSC-EV–Treg axis is emerging as a novel target for intervention in autoimmune disease (114).

### 6.3 Delivery and amplification of inflammatory mediators

EVs operate as both “carriers and amplifiers” of inflammatory cytokines. They deliver pro-inflammatory mediators such as IL-1β and TNF-α directly to distant target cells and amplify inflammatory signaling in glial cells and neurons (115). Exosomes from GD patients drive PBMC secretion of IL-6 and IL-1β through the TLR–NF-κB pathway, providing the molecular basis for systemic inflammatory dissemination (33).

EV-borne miR-155 is a pivotal miRNA linking chronic inflammation. Exosomal miR-155-5p from M1-polarized macrophages targets GDF6 and suppresses angiogenesis in a model of impaired diabetic wound healing (99). Exosomes released by hypertrophic cardiomyocytes drive IL-6 and IL-8 secretion in macrophages via the miR-155–MAPK pathway (116).

Aberrant activation of the NLRP3 inflammasome spans the spectrum of diabetic cardiovascular, renal, retinal, and neuropathic complications (117). In classical T2DM, this NLRP3-centered EV–inflammation loop, together with metabolic-stress DAMPs, constitutes the dominant molecular substrate—an important distinction from the antigen-driven T1DM axis discussed in §6.1. Under hyperglycemic stress, macrophage-derived exosomes activate glomerular mesangial cells through an NLRP3-dependent pathway and accelerate the progression of diabetic nephropathy (118). The interaction between EVs and the NLRP3 inflammasome has become a central focus in the study of diabetic complications (119). Salvia miltiorrhiza–derived exosome-like nanoparticles selectively inhibit NLRP3 inflammasome–mediated macrophage pyroptosis and offer a novel intervention strategy for diabetic cardiomyopathy (120).

Apoptotic EVs (apoEVs) transport active caspase-1, GSDMD, and DAMPs into recipient cells, triggering pyroptosis and feed-forward amplification of inflammation (121).

### 6.4 Epigenetic regulation (miRNA-mediated)

EV-loaded miRNAs constitute the core medium of intercellular epigenetic regulation by EVs. EV-miRNAs silence or activate key target genes and thereby remodel the transcriptome and functional phenotype of recipient cells (80). miR-146a functions as a key “brake-type” miRNA in the immunoregulatory network and inhibits TLR/NF-κB signaling by targeting IRAK1, IRAK2, and TRAF6 (26).

In AITD, circulating miR-15a-5p, miR-126-3p, miR-142-5p, miR-21-5p, and miR-150-5p are differentially expressed between GD and HT, and their levels correlate with thyroid hormone and anti-thyroid antibody concentrations (72). Multiple dysregulated miRNAs collectively form a core mechanism underlying HT development and progression (61).

In T1DM, T lymphocyte–derived exosomes transfer miR-142-3p, miR-142-5p, and miR-155 into pancreatic β cells, induce expression of chemokines including Ccl2, Ccl7, and Cxcl10, and trigger β-cell apoptosis (38). This EV-mediated T-cell–β-cell epigenetic network is a critical molecular mechanism in T1DM pathogenesis. EV-miRNAs also play essential roles in CD4^+^ T-cell activation, differentiation, and functional regulation (122). Across a wide range of autoimmune diseases, EV-borne miRNAs profoundly reshape immune-cell phenotypes through targeted silencing or activation of key genes (123). Standardization is critical for the translational application of EV-miRNA research. The MISEV2023 guidelines provide a systematic framework for sample handling, analytical detection, and results reporting in EV-miRNA studies (6). The convergent miRNA signatures described above are consolidated in **Table 4**, which highlights the dysregulation direction and downstream targets of recurrent EV-borne miRNAs across HT, T1DM and T2DM.

**Table 4:** Recurrent EV-borne microRNAs across HT, T1DM and T2DM and their convergent immunoregulatory functions.

miRNA	EV source	HT	T1DM	T2DM	Molecular target/pathway	Functional consequence	Ref.
miR-142-3p	T lymphocytes	↑	↑	–	RAC1/ERK1/2 ↓	Treg dysfunction; β-cell apoptosis	(8, 38)
miR-155	Macrophages, T cells, adipocytes	↑	↑	↑	PPARγ, MyoD, GDF6	Insulin resistance; impaired wound healing; chronic inflammation	(38, 39, 41, 99)
miR-146a	Multiple immune cells	↓	↓	↓	IRAK1/IRAK2/TRAF6 (TLR/NF-κB brake)	Loss of inflammatory suppression; Th17/Treg imbalance	(26, 108)
miR-21-5p	β-cells (cytokine stress)	↑ (serum EV)	↑ (3–6 fold)	↑ (DKD urinary)	Stress-response signaling	Biomarker of β-cell stress; podocyte injury in DKD	(36, 93)
miR-34a	Adipocytes	–	–	↑	Klf4 ↓	M2 macrophage suppression; adipose inflammation	(40)
miR-145-5p	Renal tubular cells (urinary EV)	–	–	↑ (DKD)	Srgap2 ↓; RhoA/ROCK ↑	Podocyte apoptosis	(92)
miR-30b-5p	Renal cells (urinary EV)	–	–	↓ (DKD)	—	Reduced; candidate diagnostic marker	(93)
miR-30b	Plasma EV	–	–	↑ (DR)	SIRT1 ↓; VEGF ↑	Proliferative diabetic retinopathy	(95)
miR-126	Circulating EV	–	–	↓	Vascular repair pathways	Microvascular and macrovascular complications	(90)
miR-20b-5p	Circulating exosomes	–	–	↑	AKTIP ↓	Skeletal-muscle insulin resistance	(75)
Let-7 family	Multiple sources	↓	–	–	Th17 differentiation (negative regulator)	Loss of tolerance maintenance	(67)

↑, upregulated; ↓, downregulated; –, not reported in the indicated disease. DKD, diabetic kidney disease; DR, diabetic retinopathy; EV, extracellular vesicle; GDF6, growth differentiation factor 6; HT, Hashimoto’s thyroiditis; IRAK1/2, interleukin-1 receptor-associated kinase 1/2; PPARγ, peroxisome proliferator-activated receptor gamma; SIRT1, sirtuin 1; T1DM, type 1 diabetes mellitus; T2DM, type 2 diabetes mellitus; Th17, T-helper 17; TLR, Toll-like receptor; TRAF6, tumor necrosis factor receptor-associated factor 6; Treg, regulatory T cell; VEGF, vascular endothelial growth factor.

## 7 Therapeutic prospects of EVs

EVs combine intrinsic nanocarrier properties—low immunogenicity, high biocompatibility, and the ability to cross multiple physiological barriers—with the capacity to be surface-engineered or cargo-loaded, positioning them as an emerging therapeutic platform for organ-specific autoimmune diseases such as HT and T1DM (124, 125). In HT and diabetes mellitus, EV-based interventions follow three principal routes: as drug-delivery carriers, as engineered immunomodulators, and as targetable nodes within EV biogenesis and uptake pathways (15–17).

Within this review we distinguish two operationally different uses of “engineered” EVs. EV-based drug delivery (§7.1) refers to EVs used primarily as passive nanocarriers of an exogenous cargo (small molecule, siRNA, insulin); the therapeutic activity resides in the loaded cargo, and modification is limited to loading and, at most, targeting-peptide display. Engineered immunomodulatory EVs (§7.2) refers to EVs whose surface or endogenous cargo has been actively reprogrammed to carry immune-instructive signals (PD-L1, Gal-9, disease-relevant miRNAs); here the therapeutic activity resides in the EV itself and its rewired signaling, not in a passive payload. Both categories require engineering steps; the distinction is which component—the payload or the vesicle—carries the intended biological effect.

### 7.1 EVs as drug-delivery carriers

EVs are an ideal platform for therapeutic delivery. Compared with synthetic nanoparticles, they originate from somatic cells, display low immunogenicity, traverse multiple biological barriers, and retain a degree of intrinsic homing capacity (124). Cargo can be incorporated by electroporation, co-incubation, sonication, or extrusion-mediated fusion, allowing the loading of small molecules, siRNAs, and miRNAs. Surface engineering via genetic fusion or chemical conjugation introduces targeting peptides that direct EVs to specific tissues or cell types (125).

In diabetes, EVs have been used to deliver multiple bioactive molecules. Insulin loaded by electroporation into exosomes derived from hepatocellular carcinoma (HepG2), dermal fibroblasts (HDFa), and pancreatic β-cells (RIN-m) enhances glucose uptake and metabolism in hyperglycemic conditions *in vitro* (126). Exosomal miR-155-5p released by M1-polarized macrophages suppresses angiogenesis through targeting GDF6, identifying one mechanism that delays diabetic wound healing; reciprocally, M2-Exo or miR-155-5p antagomiR accelerates wound closure in diabetic mice (99). For diabetic complications, MSC-derived exosomes from multiple sources mitigate renal injury in diabetic nephropathy (DN) models (127). In rat models of T1DM, conditioned medium from umbilical-cord MSCs (containing EVs) achieves both immunomodulation and β-cell regeneration (111).

Milk-derived exosomes resist degradation by gastric acid and pancreatic enzymes, making them well-suited for oral delivery. Oral administration of milk exosomes loaded with TNF-α siRNA markedly suppresses intestinal TNF-α expression in models of inflammatory bowel disease (128). Milk exosomes loaded with miR-31-5p promote angiogenesis and wound closure in diabetic mice (129).

Preclinical work directly delivering EVs to the thyroid in HT remains scarce. Most thyroid-autoimmunity studies of EVs have focused on disease mechanisms rather than therapy (20, 33), indicating substantial translational headroom.

### 7.2 Engineered EVs for immune intervention

Engineering enables EVs to actively modulate immunity. Genetic manipulation of parental cells allows EV surface display of immune-checkpoint ligands, homing peptides, or antibody fragments, while chemical modifications conjugate functional molecules directly onto EV membranes (125, 130).

In T1DM models, genetically modified macrophages secrete artificial EVs (aEVs) co-displaying PD-L1 and Gal-9. These aEVs promote apoptosis of effector T cells and Treg generation, reverse new-onset hyperglycemia in NOD mice, and reduce CD4^+^/CD8^+^ T-cell infiltration of the pancreas (131). Cytokine priming likewise elevates PD-L1 on EV surfaces and elicits immune tolerance with preservation of residual β-cell mass in T1DM models (132). β-cells themselves release PD-L1–enriched small EVs in response to inflammatory cytokines; these EVs engage PD-1 on CD8^+^ T cells and dampen cytotoxic activity, functioning simultaneously as a biomarker of disease evolution and as an endogenous protective mechanism (31).

MSC-derived EVs are the most extensively studied engineered immunomodulatory platform. In EAE, human umbilical-cord MSC exosomes upregulate Lag-3 expression on Foxp3^+^CD4^+^ Tregs and enhance their suppressive function (109). In SLE, bone-marrow MSC exosomes deliver miR-16/miR-21 that downregulate macrophage PDCD4/PTEN, drive anti-inflammatory polarization, increase Foxp3^+^ Treg infiltration, and ameliorate lupus nephritis (133). In collagen-induced arthritis, miR-146a–overexpressing MSC exosomes upregulate Foxp3, TGF-β, and IL-10 (134). Collectively, miRNA engineering of MSC-EVs reshapes Treg function across multiple autoimmune disease models.

Within AITD, tissue small EVs released by T lymphocytes carry miR-142-3p that impairs Treg function and aggravates thyrocyte destruction (8). This mechanism nominates the EV–miR-142-3p axis as an interventional target—either by blocking pathogenic miRNA delivery or by engineering EVs to deliver Treg-enhancing signals.

Clinical translation is advancing. A randomized, placebo-controlled phase II trial in patients with COVID-19–associated acute respiratory distress syndrome (ARDS) tested intravenous bone-marrow MSC-derived EVs (ExoFlo). The product was safe and well tolerated, and subgroup analyses showed reductions in 60-day mortality (135). These data provide essential safety benchmarks for the broader use of MSC-EVs in inflammatory disorders, including autoimmune disease. These findings, however, cannot be extrapolated into efficacy claims for HT or T1DM: patient etiology, dose–exposure relationships, and mechanism of action differ substantially across indications, and dedicated randomized trials in each disease remain the only acceptable evidentiary standard. The landscape of engineered and stem-cell–derived EV platforms currently under investigation for immune intervention in T1DM, T2DM and analogous autoimmune models, with implications for HT is summarized in **Table 5**.

**Table 5:** Engineered and stem-cell–derived EV strategies for immune intervention in T1DM, T2DM and analogous autoimmune models, with implications for HT.

Therapeutic platform	Cargo/surface modification	Disease model	Mechanism of action	Outcome	Reference
Artificial EVs (aEVs) from engineered macrophages	Surface PD-L1 + Gal-9	NOD mice (new-onset T1DM)	Effector T-cell apoptosis; Treg generation	Reversal of hyperglycemia; reduced pancreatic CD4^+^/CD8^+^ infiltration	(131)
Cytokine-primed EVs	Elevated PD-L1 surface	T1DM models	PD-1 engagement on CD8^+^ T cells	Immune tolerance; β-cell preservation	(132)
β-cell endogenous EVs	PD-L1 enrichment under cytokine stress	T1DM	PD-1 engagement	Dampened CD8^+^ cytotoxicity; biomarker function	(31)
K562-derived EVs	HLA-A*02/CD80/PD-L1 triplet	T1DM antigen-specific assays	Antigen-specific modulation	CD8^+^ T-cell response control	(18)
Human umbilical-cord MSC exosomes	Native miRNA cargo	EAE (autoimmune model)	Lag-3 ↑ on Foxp3^+^CD4^+^ Tregs	Enhanced Treg suppressive function	(109)
Engineered Foxp1^high^ MSC exosomes	Foxp1 overexpression	SLE	Foxp1/STAT5/Foxp3 axis	Treg reprogramming	(110)
BM-MSC exosomes	miR-16/miR-21	SLE/lupus nephritis	Macrophage PDCD4/PTEN ↓	Anti-inflammatory polarization; Treg infiltration	(133)
miR-146a–overexpressing MSC exosomes	miR-146a	Collagen-induced arthritis	Foxp3, TGF-β, IL-10 ↑	Treg enhancement	(134)
UC-MSC conditioned medium (EV-containing)	Native secretome	T1DM rat model	Treg expansion; cytokine reprogramming	Immunomodulation + β-cell regeneration	(111)
BM-MSC–derived EVs (ExoFlo)	Native	COVID-19 ARDS (Phase II RCT)	Anti-inflammatory	Safe; reduced 60-day mortality in subgroups	(135)
Milk exosomes — oral delivery	TNF-α siRNA	Inflammatory bowel disease	TNF-α suppression	Intestinal inflammation control	(128)
Milk exosomes — wound delivery	miR-31-5p	Diabetic wound healing	Angiogenesis promotion	Accelerated wound closure	(129)
HepG2/HDFa/RIN-m exosomes	Electroporated insulin	*In vitro* hyperglycemia	Enhanced glucose uptake	Insulin delivery platform	(126)
GW4869 (nSMase2 inhibitor)	Pathway blockade	EV biogenesis	Ceramide synthesis ↓	Probes EV pathogenicity	(136)
Rab27a conditional KO	Pathway blockade	Diabetic nephropathy	NF-κB tubular inflammation ↓	Slowed DN progression	(137)
FOXO1-mediated RAB27B downregulation	Endogenous regulation	Diabetic kidney	Tubular exosome secretion ↓	Reduced pathogenic EV release	(138)

↑, increase/upregulation; ↓, decrease/downregulation. Legend caveat: None of the studies listed in **Table 5** were conducted in HT models. Their inclusion in this review reflects mechanistic transferability from T1DM, other organ-specific autoimmune models (e.g. EAE, SLE, collagen-induced arthritis) and metabolic-inflammatory settings to HT, rather than direct HT-specific therapeutic evidence. This limitation is discussed in §7 and §8.3 and is aligned with the T2DM/T1DM evidence-boundary policy established in §2.3.1 and §9. Extrapolation to HT should therefore be regarded as hypothesis-generating and requires disease-matched confirmatory studies.

Retained as in the original manuscript (aEVs, ARDS, BM-MSC, DN, EAE, Foxp1/Foxp3, Gal-9, HLA-A, KO, MSC, nSMase2, PD-L1, PTEN, PDCD4, RCT, SLE, T1DM, TGF-β, TNF-α, Treg, UC-MSC).

### 7.3 Pathway-targeted interventions on EVs

Blocking the biogenesis, release, or uptake of pathogenic EVs constitutes a third therapeutic axis (136). At the biogenesis step, the neutral sphingomyelinase-2 (nSMase2) inhibitor GW4869 blocks ceramide synthesis and exosome budding, providing a standard tool for probing EV pathogenicity (136). The Rab GTPase family governs fusion of multivesicular bodies with the plasma membrane: in diabetic kidneys, FOXO1 phosphorylation downregulates RAB27B and suppresses tubular exosome secretion (138), while conditional Rab27a knockout attenuates exosome-driven NF-κB inflammatory signaling in proximal tubules and slows DN progression (137). Together, these findings identify the RAB27 family as a key node controlling EV release in diabetic complications.

At the cargo level, targeting the NLRP3 inflammasome is central to interrupting the EV–inflammation amplification loop (119). Under hyperglycemia, macrophage-derived exosomes activate the NLRP3 pathway in mesangial cells and accelerate DN (118). Apoptosis-derived EVs (apoEVs) carry active caspase-1, GSDMD, and DAMPs, triggering pyroptosis in recipient cells (121). M1 macrophage–derived EVs transfer inflammatory mitochondria into pancreatic β-cells and induce ferroptosis (32). Neutralizing or selectively clearing these danger-signal–bearing EVs represents a promising therapeutic avenue.

Mitochondrion-derived EVs occupy a distinctive niche in autoimmune initiation. They carry mitochondrial antigens and damage-associated molecules and amplify immune responses in models such as autoimmune myocarditis (106). Targeting their release pathway is emerging as a novel immunological intervention.

In AITD, circulating exosomes from GD patients are enriched in immunoregulatory proteins and activate PBMCs to secrete inflammatory cytokines (33). Building on this AITD-wide relevance, Edo et al. reported that circulating exosomes carry IGF-1R and HSP60 in Graves’ disease and engage TLR2/3–MyD88/TRIF–p65 signaling in PBMCs (139), providing a mechanistic template for probing whether analogous EV-borne pro-inflammatory ligands operate in HT and could be intercepted by neutralizing antibodies or competitive receptor peptides. This observation supports interventions using neutralizing antibodies or competitive receptor peptides to block surface proinflammatory ligands on pathogenic EVs. Proteomic studies in HT and related AITD likewise reveal differentially expressed immunoregulatory proteins on circulating exosomes that may serve as targeting candidates (20).

MDSC EVs are key regulators of immune balance. Exogenous MDSC-EVs restore Treg function in multiple inflammatory models and represent an emerging strategy for autoimmune disease intervention (114).

**Figure 4:**
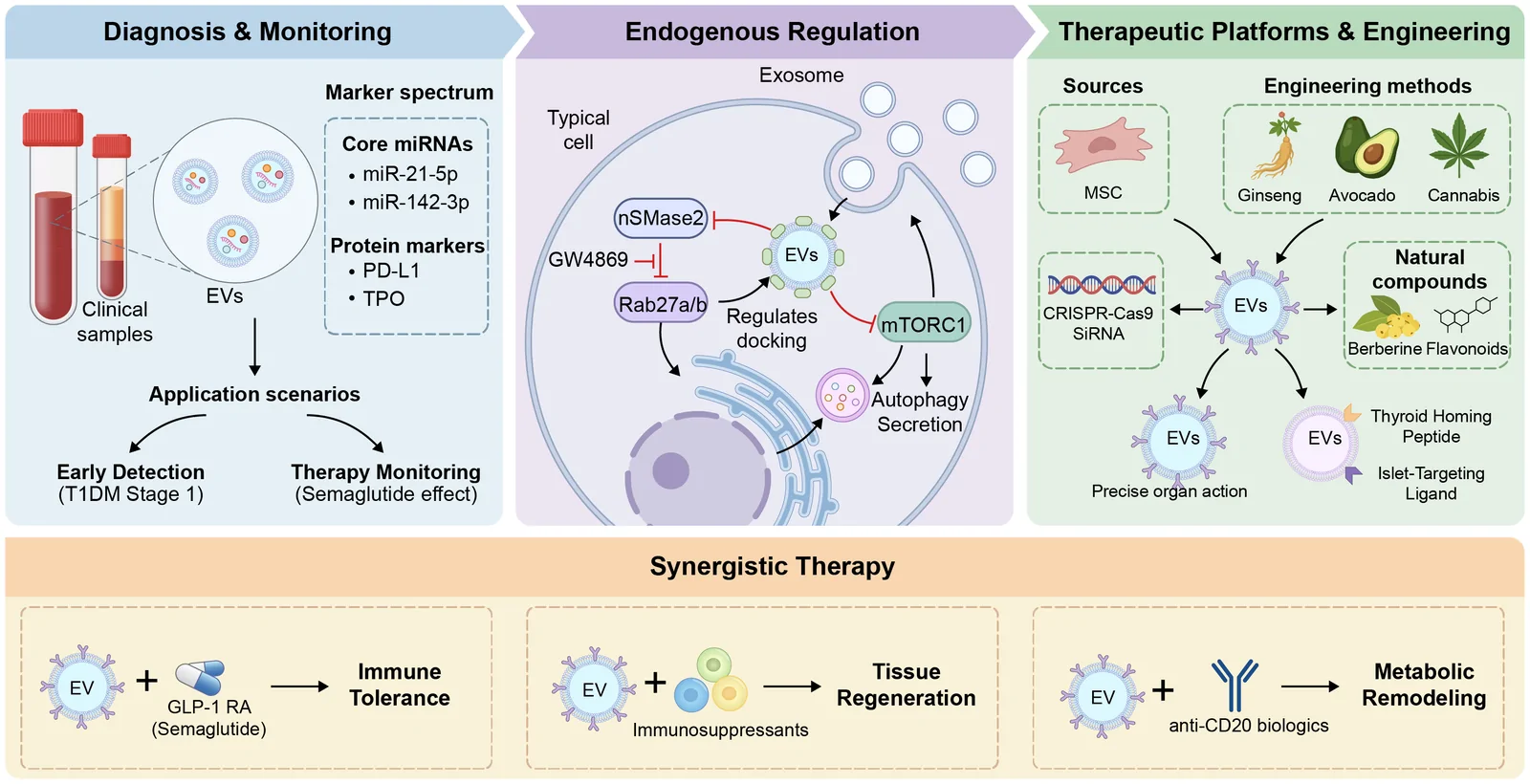
EV-based translational landscape for HT and diabetes: from diagnosis to engineered therapy. Left panel (Diagnosis and monitoring): Liquid-biopsy EVs isolated from clinical blood samples are interrogated for core miRNA markers (miR-21-5p, miR-142-3p) and protein markers (PD-L1, TPO), enabling early detection of T1DM stage 1 and longitudinal monitoring of pharmacological response (for example, semaglutide therapy). Center panel (Endogenous regulation): EV biogenesis and release are governed by the nSMase2 ceramide pathway (pharmacologically inhibited by GW4869) and the Rab27a/b–mediated multivesicular-body–plasma-membrane docking machinery; mTORC1 signaling reciprocally regulates autophagy-coupled EV secretion, offering druggable nodes for therapeutic modulation. Right panel (Therapeutic platforms and engineering): EV sources span mesenchymal stem cells, plant-derived exosome-like nanoparticles (PELNs from ginseng, avocado and cannabis), and synthetic natural-compound carriers (berberine, flavonoids). CRISPR-Cas9 and siRNA loading, together with surface decoration using thyroid-homing peptides or islet-targeting ligands, enable precise organ-directed delivery. Bottom panel (Synergistic therapy): EV-based interventions can be combined with GLP-1 RA (semaglutide) for immune-tolerance restoration, with immunosuppressants for tissue regeneration, or with anti-CD20 biologics for metabolic remodeling. CD20, cluster of differentiation 20 (B-cell surface antigen); CRISPR-Cas9, clustered regularly interspaced short palindromic repeats/CRISPR-associated protein 9; EVs, extracellular vesicles; GLP-1 RA, glucagon-like peptide-1 receptor agonist; GW4869, neutral sphingomyelinase-2 inhibitor; HT, Hashimoto’s thyroiditis; miRNA, microRNA; MSC, mesenchymal stem cell; mTORC1, mechanistic target of rapamycin complex 1; nSMase2, neutral sphingomyelinase 2; PD-L1, programmed death-ligand 1; PELNs, plant-derived exosome-like nanoparticles; Rab27a/b, Ras-related protein Rab-27A/Rab-27B; siRNA, small interfering RNA; T1DM, type 1 diabetes mellitus; TPO, thyroid peroxidase.

Together, non-invasive EV diagnostics, modulation of endogenous EV biogenesis, and engineered EV delivery platforms define the main translational routes for HT, T1DM and T2DM (**Figure 4**). Combination strategies with established pharmacotherapies remain plausible, but they still require disease-matched validation.

Two overarching cautions govern the therapeutic landscape summarized above. First, in organ-specific autoimmune diseases such as HT and T1DM, non-selective EV-mediated immunomodulation risks aggravating rather than restoring immune tolerance, because indiscriminate suppression may license off-target autoreactivity elsewhere; selective delivery to the thyroid or islet compartment—not systemic immunosuppression—must therefore be the therapeutic goal. Second, engineered EVs still face critical translational bottlenecks: batch-to-batch consistency of biogenesis and cargo loading, *in vivo* clearance kinetics that constrain therapeutic dosing windows, and the risk of neutralizing-antibody induction against surface-displayed immunomodulatory proteins on repeat administration. Under these constraints, existing phase II safety data (e.g. ExoFlo, ref 136) function as translational benchmarks rather than efficacy signals, and no HT- or T1DM-specific efficacy claim should be inferred until dedicated randomized trials are completed.

## 8 Challenges and future perspectives

Although substantial progress has been made in characterizing EV-mediated immune regulation in HT and diabetes, clinical translation remains constrained by technical, biological, and manufacturing challenges that span the entire EV research pipeline. Rather than enumerating these limitations in isolation, this chapter integrates current bottlenecks with their corresponding solution trajectories along three interconnected dimensions — methodological standardization, model fidelity, and biomarker–therapeutic translation — before consolidating them into a forward-looking framework. Building on the therapeutic landscape outlined in Section 7, the analysis below extends the same evidence-boundary discipline: mechanistic transferability from T1DM and analogous autoimmune models to HT is treated as a working hypothesis, not as established HT-specific efficacy.

### 8.1 Methodological bottlenecks: from isolation heterogeneity to single-vesicle resolution

The most upstream obstacle to reproducible EV research lies in isolation and characterization. EVs share overlapping physicochemical properties with lipoproteins and free protein aggregates, and yield, purity, and subpopulation composition differ markedly among differential ultracentrifugation, density-gradient ultracentrifugation, size-exclusion chromatography, polyethylene glycol precipitation, and immunoaffinity capture (140). No single method currently balances yield, purity, reproducibility, and scalability (141). Although the MISEV2023 guidelines specify minimum reporting standards for EV source, isolation workflow, and characterization parameters (6), a systematic review of 471 registered EV clinical trials documented that most studies failed to report isolation methods or subpopulation definitions, with subpopulation disclosure below 30% (142); a parallel meta-analysis of 21 clinical studies found that only three explicitly followed the MISEV framework, rendering cross-trial comparisons of efficacy and safety unreliable (143).

Compounding the isolation problem is intrinsic EV heterogeneity. Subpopulations from the same parental cell can carry opposing biological signals, and bulk-level analyses obscure this divergence, preventing attribution of biological effects to specific pathogenic subsets (7, 144). *In vivo* tracking faces an analogous resolution gap: lipophilic dyes such as the PKH series readily form micellar aggregates that distort particle-size distributions and generate false-positive signals (145), while EVs coexist with free miRNA–AGO2 complexes and lipoprotein particles in biofluids, making functional attribution to disease-associated EVs particularly difficult (6, 13).

The methodological response to these compounded bottlenecks is converging on single-vesicle multi-omics. Imaging flow cytometry, single-particle interferometric reflectance imaging, single-vesicle RNA sequencing, and digital ELISA already permit resolution at the individual-particle level (144), and their integration with super-resolution imaging, microfluidic sorting, and machine-learning classifiers offers a realistic path toward identifying HT- and diabetes-specific subpopulation signatures and predicting disease activity (144). Coupling these analytical engines with international enforcement of MISEV2023 reporting — including unified minimum metrics for EV concentration, purity, subpopulation ratio, and cargo integrity (6) — represents the most tractable near-term route to reproducible EV science.

### 8.2 Model fidelity: bridging the rodent-to-human translational gap

Mechanistic studies of EV-mediated autoimmune and metabolic–immune regulation rely heavily on cell culture and rodent models, both of which only partially recapitulate human disease. The NOD mouse remains the canonical T1DM model, yet its disease kinetics, autoantigen repertoire, and insulin secretion profile differ substantively from human T1DM (13). Thyroid EV research faces equivalent constraints, as existing induced or spontaneous thyroiditis models reproduce only fragments of the chronic lymphocytic infiltration that defines human HT (14). Mesenchymal stem cell (MSC)–derived EVs restore immune tolerance and protect β cells in preclinical T1DM models, but translation to the complex human immune background requires large-scale validation (17).

Patient-derived humanized platforms offer a more realistic way to study disease-relevant immune–target-cell interactions than conventional rodent or monoculture systems. Patient-derived induced pluripotent stem cell (iPSC)–differentiated islet organoids co-cultured with autologous immune cells more faithfully reproduce the autoimmune attack of T1DM and provide a tractable platform for testing EV-mediated immune regulation and therapeutic interventions (13). Analogous thyroid organoid systems — from the seminal demonstration that mouse embryonic stem cells can be directed toward functional thyroid follicular epithelium (146), through the derivation of thyroid progenitors from human pluripotent stem cells (147), to the more recent generation of iPSC-derived thyroid organoids that recapitulate follicular architecture and hormone synthesis (148) — now provide a realistic HT-side analogue. When paired with patient-derived immune cells, these models can narrow the translational gap on the HT side and can be integrated with single-vesicle multi-omics for biomarker and therapeutic studies.

### 8.3 Biomarker validation and manufacturing: from discovery to GMP-grade deployment

Two practical problems now dominate translational EV research: how to validate EVs as liquid-biopsy biomarkers, and how to manufacture engineered EVs as reproducible therapeutics. In both settings, candidate biomarkers and therapeutic products remain largely confined to single-center, small-sample exploratory studies (16), while variation in parental cell selection, passage number, culture conditions, scale-up, storage, and stability threatens lot consistency and clinical-grade reproducibility (149). Surface molecules and endogenous cargo can also trigger host immune responses, exposing engineered EVs to rapid macrophage clearance and neutralizing antibody induction *in vivo* (150). Clinical-trial analyses likewise show no consensus on optimal dose range, dosing frequency, or route of administration, leaving dose–response relationships poorly defined (142).

On the biomarker side, T lymphocyte–derived EVs enriched in miR-142-3p mediate Treg dysfunction and thyrocyte destruction in HT (8), β-cell–derived EVs carrying PD-L1 are a candidate biomarker for the evolution of T1DM (31), and circulating EV-miRNA profiles contribute to metabolic–immune crosstalk in T2DM without implying an autoantigen-driven mechanism in classical T2DM (9). Advancing these candidates requires multicenter, large-cohort prospective studies with rigorous diagnostic-accuracy assessment and longitudinal follow-up (15). On the therapeutic side, artificial EVs presenting PD-L1 and galectin-9 reduce pancreatic T-cell infiltration and ameliorate hyperglycemia in new-onset T1DM NOD mice (131), K562-derived EVs presenting an HLA-A*02/CD80/PD-L1 triplet enable antigen-specific modulation of CD8^+^ T-cell responses (18), and MSC-derived EVs restore immune tolerance and protect β cells in preclinical T1DM models (17). The next stage must transition these constructs from proof-of-concept to investigational new drug (IND) submission and Phase I/II trial design, supported by GMP-compliant upstream and downstream processes — clear parental cell traceability, lot-to-lot consistency, defined quality control — aligned with ISEV, FDA, and EMA regulatory frameworks (149). The principal bottlenecks across the EV research pipeline — together with the corresponding translational pathways outlined in Sections 8.1–8.3 — are consolidated in **Table 6**.

**Table 6:** Current bottlenecks and corresponding translational pathways for EV research in HT and diabetes.

Dimension	Current bottleneck	Key evidence	Translational pathway	Reference
Isolation & purification	No single method balances yield/purity/scalability; overlap with lipoproteins and aggregates	<30% of 471 registered EV trials report subpopulation definitions; only 3/21 follow MISEV	International enforcement of MISEV2023 minimum reporting standards	(6, 140–143)
Subpopulation heterogeneity	Bulk analyses obscure opposing subpopulation signals	Same parental cell releases functionally divergent EVs	Single-vesicle multi-omics (imaging flow cytometry, single-particle interferometry, single-vesicle RNA-seq, digital ELISA) integrated with machine learning	(7, 144)
*In vivo* tracking	PKH dyes form micellar aggregates; free miRNA–AGO2 and lipoproteins confound attribution	False-positive size distributions; ambiguous functional attribution	Non-aggregating labeling chemistries; orthogonal *in situ* tracking	(6, 13, 145)
Model fidelity	NOD mice and induced thyroiditis models incompletely recapitulate human disease	Divergent kinetics, autoantigen repertoire, and infiltration pattern	Patient-derived iPSC islet/thyroid organoids + autologous immune co-culture	(13, 14, 17)
Biomarker validation	Single-center, small-sample exploratory studies dominate	miR-142-3p (HT), β-cell EV PD-L1 (T1DM), EV-miRNA panels (T2DM) candidates pending	Multicenter prospective cohorts with rigorous diagnostic accuracy and longitudinal follow-up	(8, 9, 15, 16, 31)
Manufacturing & immunogenicity	Lot variability; rapid macrophage clearance; neutralizing antibody induction	No consensus on dose, frequency, route in clinical trials	GMP-compliant upstream/downstream processes; ISEV–FDA–EMA-aligned IND submission	(142, 149, 150)
Therapeutic translation	Engineered EVs remain at preclinical stage in autoimmune endocrine disease	aEVs (PD-L1/Gal-9), K562 HLA-A*02/CD80/PD-L1, MSC-EVs validated preclinically	Phase I/II trial design under regulatory frameworks	(17, 18, 131, 149)

aEVs, artificial extracellular vesicles; AGO2, Argonaute 2; EMA, European Medicines Agency; EV, extracellular vesicle; FDA, U.S. Food and Drug Administration; GMP, good manufacturing practice; HT, Hashimoto’s thyroiditis; IND, investigational new drug; iPSC, induced pluripotent stem cell; ISEV, International Society for Extracellular Vesicles; MISEV2023, Minimal Information for Studies of Extracellular Vesicles 2023; MSC, mesenchymal stem cell; NOD, non-obese diabetic; PD-L1, programmed death-ligand 1; PKH, lipophilic dye family; T1DM, type 1 diabetes mellitus; T2DM, type 2 diabetes mellitus.

A cautionary framing is warranted before advancing any of these constructs toward organ-specific autoimmune indications such as HT. First, in organ-specific autoimmune disease, non-selective immunomodulatory EVs may amplify off-target immune activation rather than restore tolerance; therapeutic intent must therefore be anchored to defined antigen specificity or to lineage-restricted immunoregulatory cargo, not to generic “MSC-EV immunosuppression”. Second, batch-to-batch consistency, *in vivo* clearance kinetics and the induction of neutralizing antibodies against engineered EV surfaces remain unresolved manufacturing bottlenecks that will directly limit dose repeatability in chronic autoimmune conditions requiring long-term administration. Third, the Phase II safety benchmark provided by exosome-based interventions such as ExoFlo in acute inflammatory contexts (135) should be read strictly as a safety benchmark for that indication; it does not license extrapolation of efficacy to HT or T1DM, where the target immune circuitry, chronicity and endpoint definitions differ fundamentally. Positioning engineered EV therapeutics against HT and diabetes therefore requires disease-matched safety and efficacy trials, with pre-specified immune-monitoring endpoints (autoantibody titers, thyroid-infiltrating lymphocyte signatures, β-cell function metrics) rather than reliance on cross-indication analogy.

### 8.4 Convergent outlook

**Figure 5:**
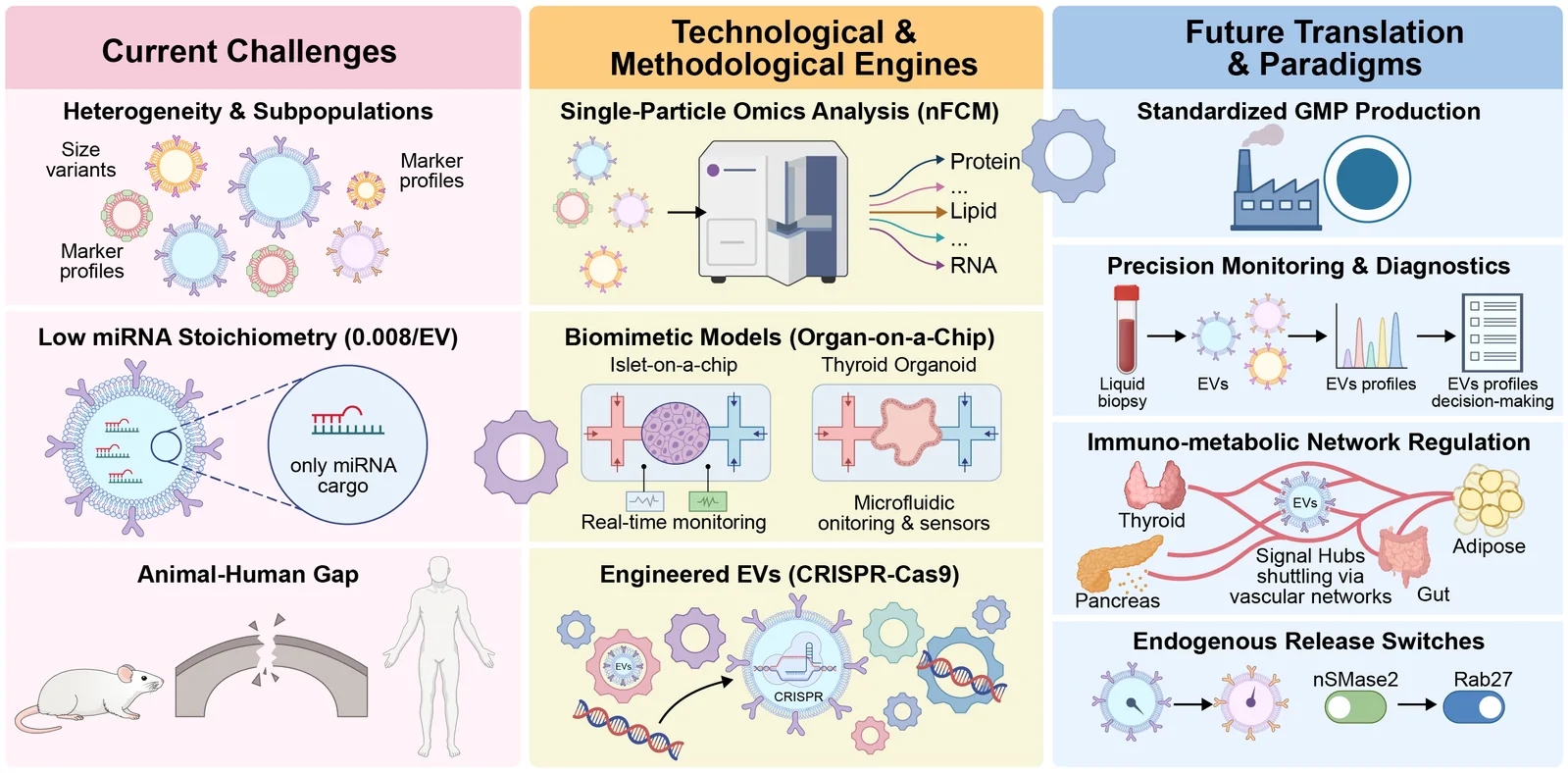
Current bottlenecks, enabling technologies and future translational paradigms for EV research in HT and diabetes. Left column (Current challenges): Three core limitations restrict clinical translation — (i) marked heterogeneity of EV subpopulations differing in size, surface marker profile and cargo composition; (ii) extremely low miRNA stoichiometry (approximately 0.008 copies per EV) that complicates quantitative biology; and (iii) the species gap between rodent models and human disease. Middle column (Technological and methodological engines): Single-particle multi-omics analysis using nano-flow cytometry (nFCM) enables simultaneous protein, lipid and RNA profiling at single-vesicle resolution. Biomimetic organ-on-a-chip models — islet-on-a-chip and thyroid organoids — provide physiologically faithful platforms for functional EV assays coupled with real-time microfluidic monitoring. CRISPR-Cas9–based EV engineering allows rational cargo programming. Right column (Future translation and paradigms): Standardized GMP-compliant EV production, precision EV-based liquid biopsy informing clinical decision-making, mapping of the inter-organ immuno-metabolic EV signaling network spanning thyroid, pancreas, gut and adipose tissue, and pharmacological control of endogenous EV release through nSMase2 and Rab27 switches as next-generation druggable targets. CRISPR-Cas9, clustered regularly interspaced short palindromic repeats/CRISPR-associated protein 9; EVs, extracellular vesicles; GMP, good manufacturing practice; HT, Hashimoto’s thyroiditis; MISEV2023, Minimal Information for Studies of Extracellular Vesicles 2023; miRNA, microRNA; nFCM, nano-flow cytometry; nSMase2, neutral sphingomyelinase 2; Rab27, Ras-related protein Rab-27.

Single-vesicle multi-omics, humanized organoid models, and GMP-grade standardization form a connected translational pipeline for EV biomarker discovery, product characterization, and therapeutic testing (**Figure 5**). Coupled with CRISPR-Cas9–based EV cargo engineering, this pipeline may narrow current bottlenecks, but deployment must remain calibrated to the evidence boundary established in this review: EV-based autoantigen-directed strategies are best aligned with HT and T1DM, whereas EV-based interventions in T2DM should target metabolic–inflammatory circuitry rather than autoantigen presentation.

## 9 Conclusion

EVs are emerging as important mediators of immune-microenvironment remodeling across HT and diabetes mellitus. In HT and T1DM, the strongest evidence supports EV-mediated autoantigen transfer together with dysregulated T-cell and antigen-presenting-cell crosstalk; in classical T2DM, EVs are better supported as carriers of stress signals, inflammatory chemokines, and immunoregulatory miRNAs that sustain chronic metabolic inflammation rather than autoantigen presentation. These differences matter for translation: advances in MISEV2023-guided characterization, single-vesicle multi-omics, humanized models, and manufacturing standardization may improve biomarker development and therapeutic design, but disease-matched validation remains essential before EV-based strategies can be applied confidently in HT or diabetes.

## Glossary

ADCC: antibody-dependent cellular cytotoxicity
aEVs: apoptotic/artificial extracellular vesicles
AGEs: advanced glycation end-products
AGO2: Argonaute 2
AITD: autoimmune thyroid disease
AKT: protein kinase B
Alix: ALG-2-interacting protein X
ARDS: acute respiratory distress syndrome
ATMs: adipose tissue macrophages
BM-MSC: bone marrow–derived mesenchymal stem cell
CD4⁺/CD8⁺: cluster of differentiation 4/8 positive T cell
CD9/CD63/CD81: tetraspanin exosome markers
CD20: cluster of differentiation 20 (B-cell surface antigen)
CD40/CD83: co-stimulatory molecules on dendritic cells
CRISPR-Cas9: clustered regularly interspaced short palindromic repeats/CRISPR-associated protein 9
CXCL10: C-X-C motif chemokine ligand 10
DAMPs: damage-associated molecular patterns
DC/DCs: dendritic cell(s)
DKD: diabetic kidney disease
DN: diabetic nephropathy
DPN: diabetic peripheral neuropathy
DR: diabetic retinopathy
EAE: experimental autoimmune encephalomyelitis
ELISA: enzyme-linked immunosorbent assay
EMA: European Medicines Agency
EMPs: endothelial microparticles
ESCRT: endosomal sorting complex required for transport
EVs: extracellular vesicles
FDA: Food and Drug Administration
Foxp1/Foxp3: forkhead box P1/P3
GAD65: glutamic acid decarboxylase 65
Gal-9: galectin-9
GD: Graves’
GLP-1 RA: glucagon-like peptide-1 receptor agonist
GMP: good manufacturing practice
GO: Graves’
GW4869: neutral sphingomyelinase-2 inhibitor
gWAT: gonadal (visceral) white adipose tissue
HA: hyaluronic acid
HbA1c: glycated hemoglobin
HLA-A: human leukocyte antigen A
HMGB1: high-mobility group box 1
HSP60: heat shock protein 60
HT: Hashimoto’s thyroiditis
IA-2: islet antigen 2
IDF: International Diabetes Federation
IFN-γ: interferon gamma
IL-1β/IL-4/IL-6/IL-8/IL-10/IL-17: interleukin 1β/4/6/8/10/17
IND: investigational new drug
iPSC: induced pluripotent stem cell
IRAK1/2: interleukin-1 receptor-associated kinase 1/2
IRS1: insulin receptor substrate 1
ISEV: International Society for Extracellular Vesicles
JAK/STAT: Janus kinase/signal transducer and activator of transcription
KO: knockout
LADA: latent autoimmune diabetes in adults
M1/M2: classically (pro-inflammatory)/alternatively activated macrophage phenotype
MAFLD: metabolic dysfunction-associated fatty liver disease
MDSCs: myeloid-derived suppressor cells
MHC-II: major histocompatibility complex class II
miRNA: microRNA
MISEV2023: Minimal Information for Studies of Extracellular Vesicles 2023
MSC: mesenchymal stem cell
MSC-EVs: mesenchymal stem cell–derived extracellular vesicles
mTORC1: mechanistic target of rapamycin complex 1
MVB(s): multivesicular body/bodies
NF-κB: nuclear factor kappa B
nFCM: nano-flow cytometry
NLRP3: NLR-family pyrin domain containing 3
NOD: non-obese diabetic (mouse)
nSMase2: neutral sphingomyelinase 2
OFs: orbital fibroblasts
OMVs: outer membrane vesicles
PBMCs: peripheral blood mononuclear cells
PD-L1: programmed death-ligand 1
PDCD4: programmed cell death 4
PDMPs: platelet-derived microparticles
PELNs: plant-derived exosome-like nanoparticles
PI3K: phosphoinositide 3-kinase
PKH: lipophilic dye family (for EV labeling)
PPARγ: peroxisome proliferator-activated receptor gamma
PTEN: phosphatase and tensin homolog
Rab27a/b: Ras-related protein Rab-27A/Rab-27B
RAGE: receptor for advanced glycation end-products
RCT: randomized controlled trial
ROS: reactive oxygen species
SASP: senescence-associated secretory phenotype
siRNA: small interfering RNA
SLE: systemic lupus erythematosus
T1DM: type 1 diabetes mellitus
T2DM: type 2 diabetes mellitus
TFCs: thyroid follicular cells
Tfh: T follicular helper cell
TGF-β/TGF-β1: transforming growth factor beta/beta 1
Th: T-helper (cell)
Th1/Th17: T-helper 1/17 cell
TLR: Toll-like receptor
TNF-α: tumor necrosis factor alpha
TPO: thyroid peroxidase
Tph: T peripheral helper cell
TRAb: thyrotropin receptor antibody
TRAF6: tumor necrosis factor receptor-associated factor 6
Treg/Tregs: regulatory T cell(s)
TSG101: tumor susceptibility gene 101
TSHR: thyrotropin (thyroid-stimulating hormone) receptor
UC-MSC: umbilical cord–derived mesenchymal stem cell
VEGF: vascular endothelial growth factor
ZnT8: zinc transporter 8.
